# Supplementary material for: Absolute Configurations and Chitinase Inhibitions of Quinazoline-Containing Diketopiperazines from the Marine-Derived Fungus Penicillium polonicum
Source: Mar Drugs. 2020 Sep 21;18(9):479. doi: 10.3390/md18090479 (PMC7551877; doi:10.3390/md18090479)
Supplement: Supplementary file 1 [file marinedrugs-18-00479-s001.pdf]

marine drugs

Xing-Chen Guo [1,†], Ya-Hui Zhang [1,†], Wen-Bin Gao [2], Li Pan [3], Hua-Jie Zhu [1,*] and Fei Cao [1,*]

[1] College of Pharmaceutical Sciences, Institute of Life Science and Green Development, Key Laboratory of Medicinal Chemistry and Molecular Diagnosis of Ministry of Education, Hebei University, Baoding 071002, China; guoxingchen92@163.com (X.-C.G.); 15689932652@163.com (Y.-H.Z.)
[2] Coollege of Life Science, Cangzhou Normal University, Cangzhou 061001, China; wenbinxing@yeah.net
[3] State Key Laboratory of NBC Protection for Civilian, Beijing, 102205, China; bk6180b@163.com
* Correspondence: hjzhu2019@163.com (H.-J.Z.); caofei542927001@163.com (F.C.)
† These authors contributed equally to this work.

List of Supporting Information

Figure S1. ^{1}H NMR (600 MHz, DMSO-d_6) spectrum of **1**.

Figure S2. ^{13}C NMR (150 MHz, DMSO-d_6) spectrum of **1**.

Figure S3. HSQC (DMSO-d_6) spectrum of **1**.

Figure S4. ^{1}H-^{1}H COSY (DMSO-d_6) spectrum of **1**.

Figure S5. HMBC (DMSO-d_6) spectrum of **1**.

Figure S6. HRESIMS for compound **1**.

Figure S7. ^{1}H NMR (600 MHz, DMSO-d_6) spectrum of **2**.

Figure S8. ^{13}C NMR (150 MHz, DMSO-d_6) spectrum of **2**.

Figure S9. HSQC (DMSO-d_6) spectrum of **2.**

Figure S10. ^{1}H-^{1}H COSY (DMSO-d_6) spectrum of **2.**

Figure S11. HMBC (DMSO-d_6) spectrum of **2.**

Figure S12. 1D NOE (DMSO-d_6) spectrum of compounds **1** and **2**.

Figure S13. HRESIMS for compound **2.**

Figure S14. ^{1}H NMR (600 MHz, DMSO-d_6) spectrum of **3**.

Figure S15. ^{1}H NMR (600 MHz, $CDCl_3$) spectrum of **3**.

Figure S16. ^{13}C NMR (150 MHz, DMSO-d_6) spectrum of **3**.

Figure S17. HSQC (DMSO-d_6) spectrum of **3.**

Figure S18. ^{1}H-^{1}H COSY (DMSO-d_6) spectrum of **3.**

Figure S19. HMBC (DMSO-d_6) spectrum of **3.**

Figure S20. NOESY ($CDCl_3$) spectrum of compound **3.**

Figure S21. HRESIMS for compound **3.**

Figure S22. ^{1}H NMR (600 MHz, DMSO-d_6) spectrum of **4**.

Figure S23. ^{1}H NMR (600 MHz, DMSO-d_6) spectrum of **5**.

Figure S24. ^{1}H NMR (600 MHz, DMSO-d_6) spectrum of **6**.

Figure S25. ^{1}H NMR (600 MHz, DMSO-d_6) spectrum of **7**.

Figure S26. Experimental ECD spectra of **1–5**.

Figure S27. Experimental and calculated ECD spectra of **1**.

Figure S28. Experimental and calculated ECD spectra of **2**.

Figure S29. Experimental and calculated ECD spectra of **3**.

Figure S30. Experimental and calculated ECD spectra of **4**.

Figure S31. Experimental and calculated ECD spectra of **5**.

Figure S32. The data of DP4plus method of compound **3** (B3lyp/6-311+G(d,p))

Figure S33. The data of DP4plus method of compound **5** (B3lyp/6-311+G(d,p))

Figure S34. Compound **1** tightly bind to the entire active pocket of *Of*Chi-h

Figure S35. Three hydrogen bonds formed by **1** with the guanidine group of ARG439

Figure S36. π-Sulfur interaction between **1** and the sulfur atom of methionine MET381

Figure S37. π-π Stacking between **1** and the benzene rings of Trp268 and Phe309

Figure S38. Alkyl hydrophobic interactions between **1** and Ala355/Met381, and between **1** and Val469

Figure S39. Mixed π/alkyl hydrophobic interactions between **1** and Tyr156, and between **1** and Phe184

Figure S40. Compound **4** tightly bind to the *Of*Hex1 in a "U" conformation

Figure S41. Hydrogen bonds formed between **4** and the guanidine group of ARG220, and between **4** and the carboxyl of ASP367

Figure S42. Compound **4** had a π-anion with the carboxyhydroxyl oxygen anion in the residue of GLU368

Figure S43. π-π Stacking interaction between **4** and the indole ring of Trp490

Figure S44. Alkyl hydrophobic interaction between **4** and the isopropyl group of Val484

Figure S45. Mixed π/alkyl hydrophobic interactions between **4** and Trp322/Trp483, and between **4** and the isopropyl group of Val327

Figure S46. N-H hydrogen bond interaction between **2** and Arg220

Table S1. Comparison of the data of DP4plus method of compound **3** and **5**

Table S2. Cytotoxic activity data of compounds **1–7**.

Table S3. The coordinate for the lowest-energy conformer of compound **1** for ECD calculation.

Table S4. The coordinate for the lowest-energy conformer of compound **2** for ECD calculation.

Table S5. The coordinate for the lowest-energy conformer of compound **3** for ECD calculation.

Table S6. The coordinate for the lowest-energy conformer of compound **4** for ECD calculation.

Table S7. The coordinate for the lowest-energy conformer of compound **5** for ECD calculation.

Table S8-S16. The coordinates for the conformers of compound **3** for NMR calculation.

Table S17-S32. The coordinates for the conformers of compound **5** for NMR calculation.

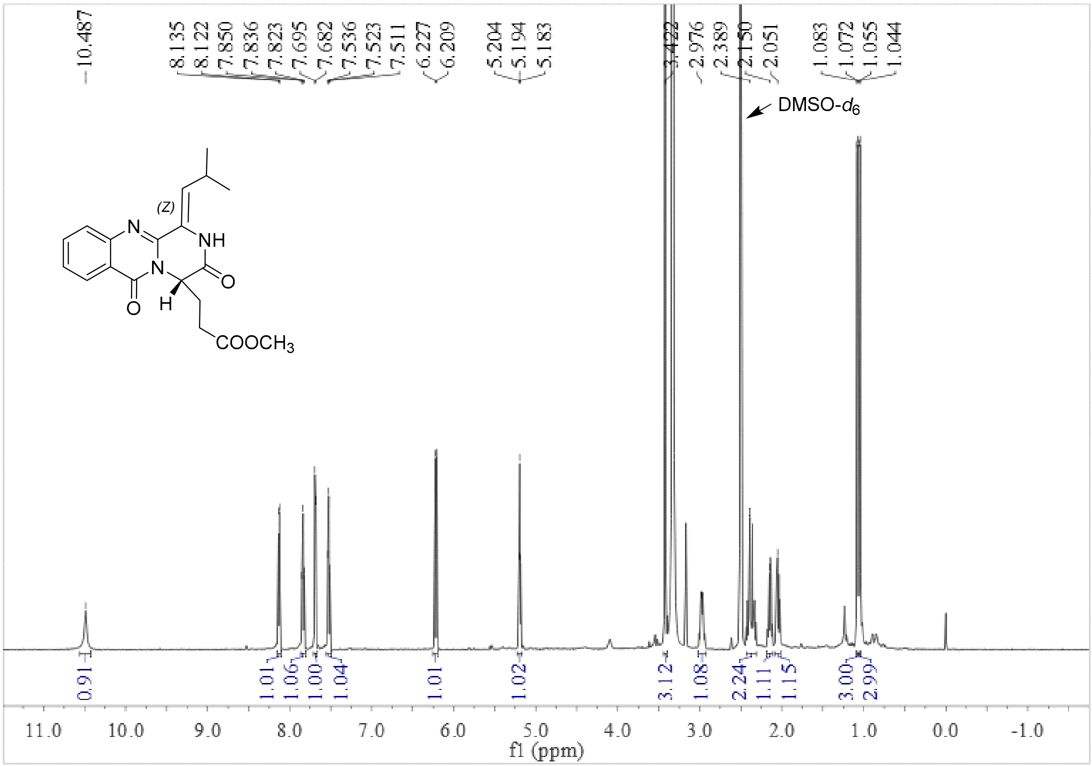

Figure S1. ^{1}H NMR (600 MHz, DMSO-d_6) spectrum of **1**.

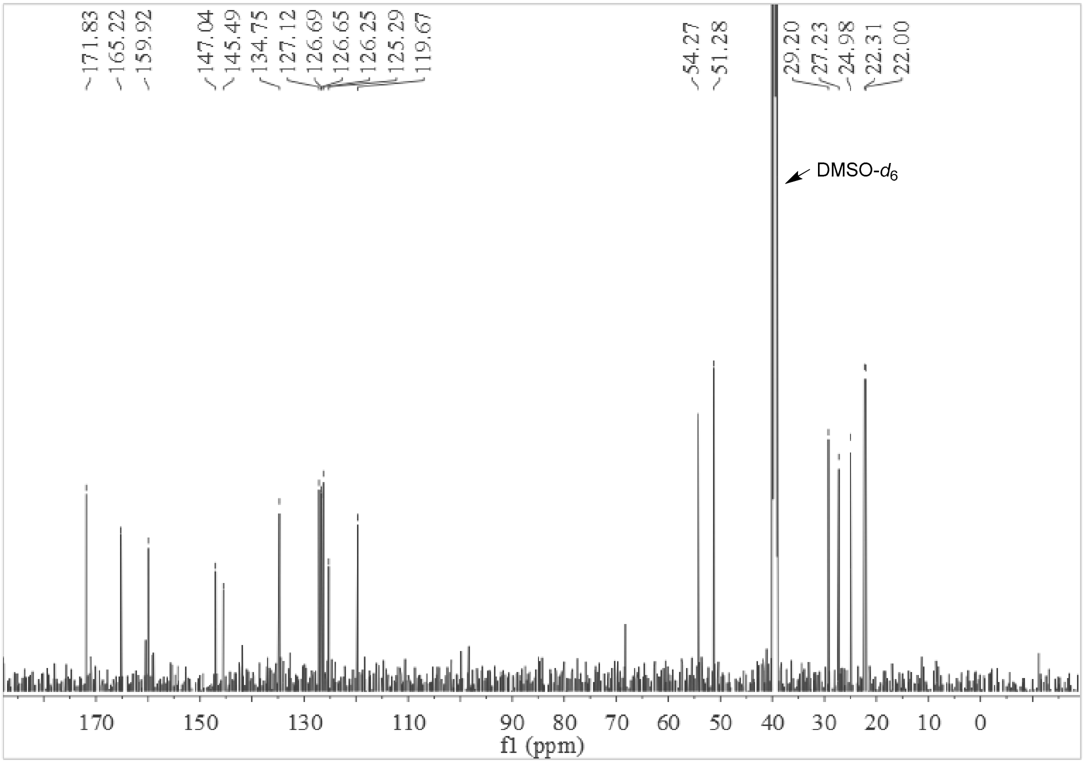

Figure S2. ^{13}C NMR (150 MHz, DMSO-d_6) spectrum of compound **1.**

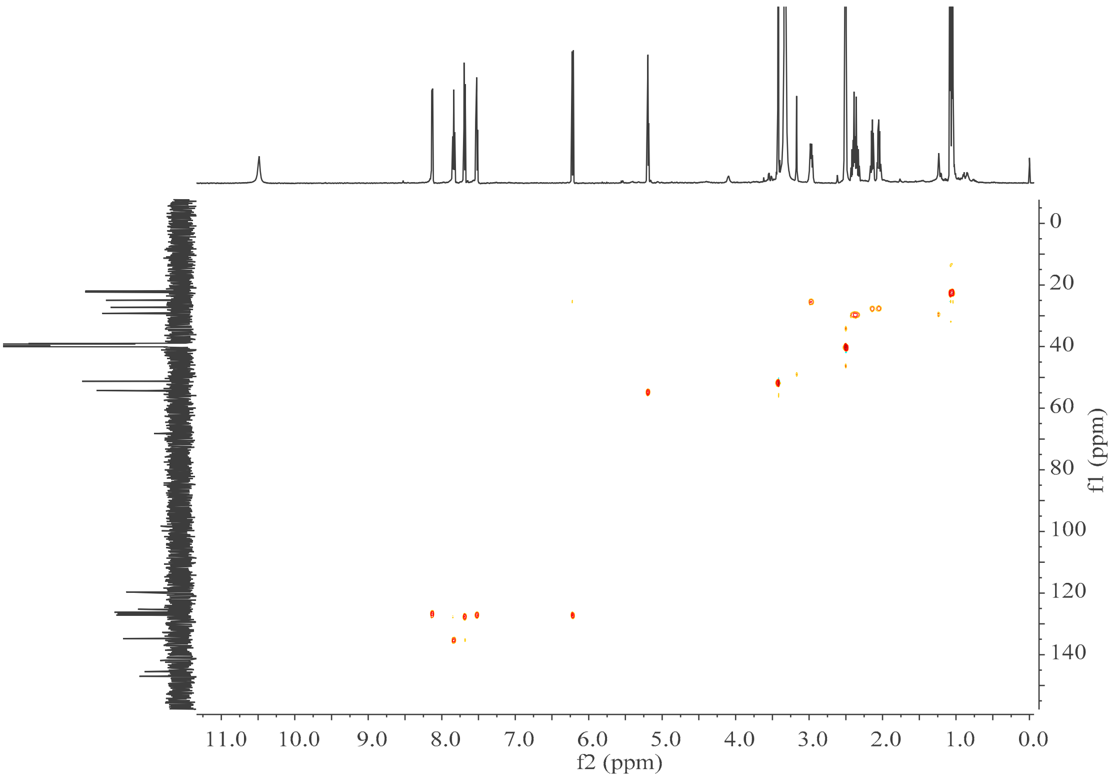

Figure S3. HSQC (DMSO-d_6) spectrum of **1**.

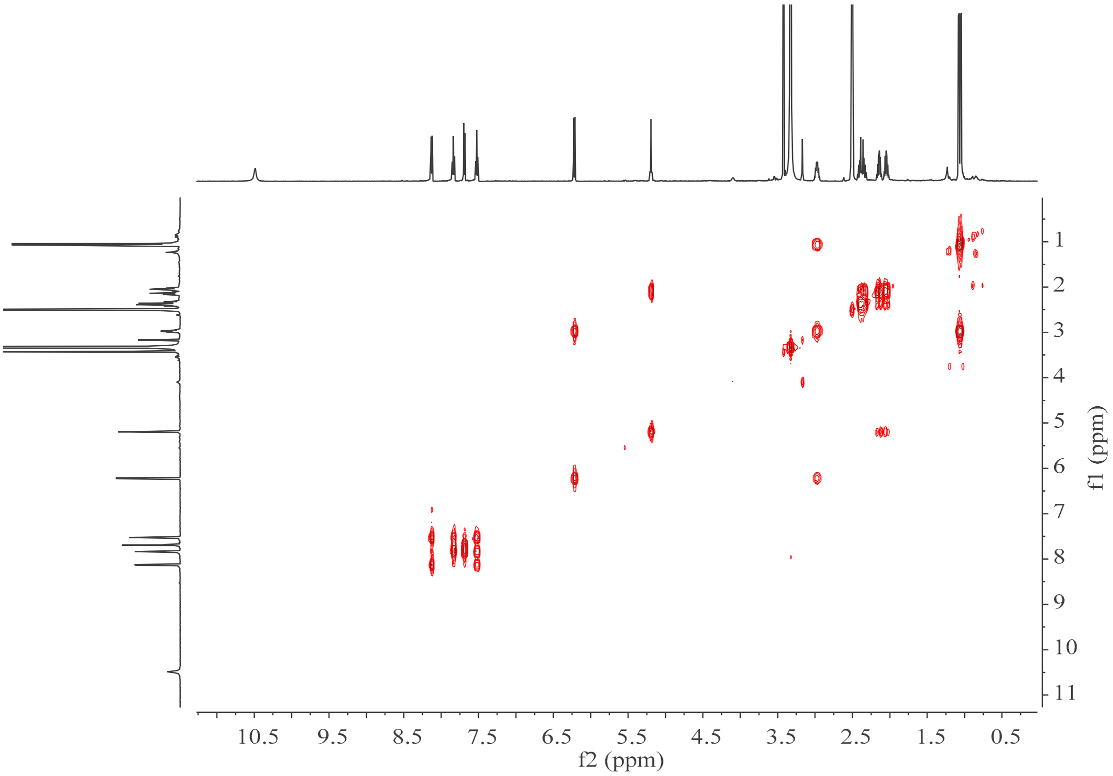

Figure S4. ^{1}H-^{1}H COSY (DMSO-d_6) spectrum of **1**.

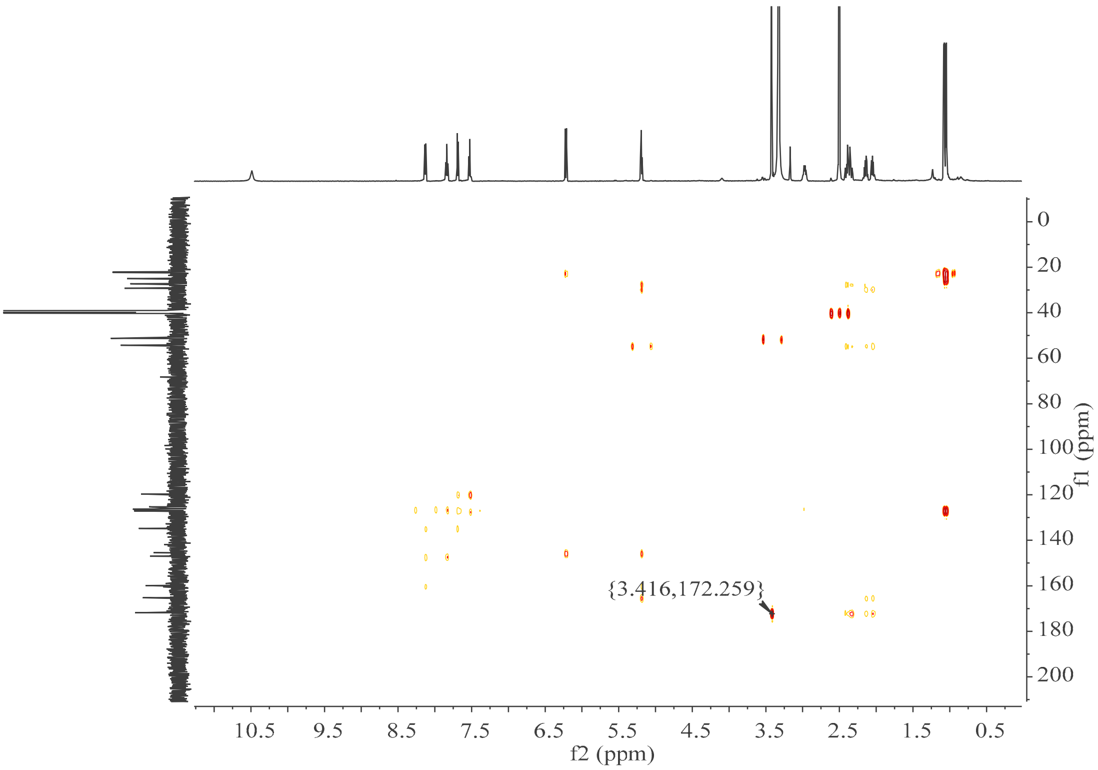

Figure S5. HMBC (DMSO-d_6) spectrum of **1**.

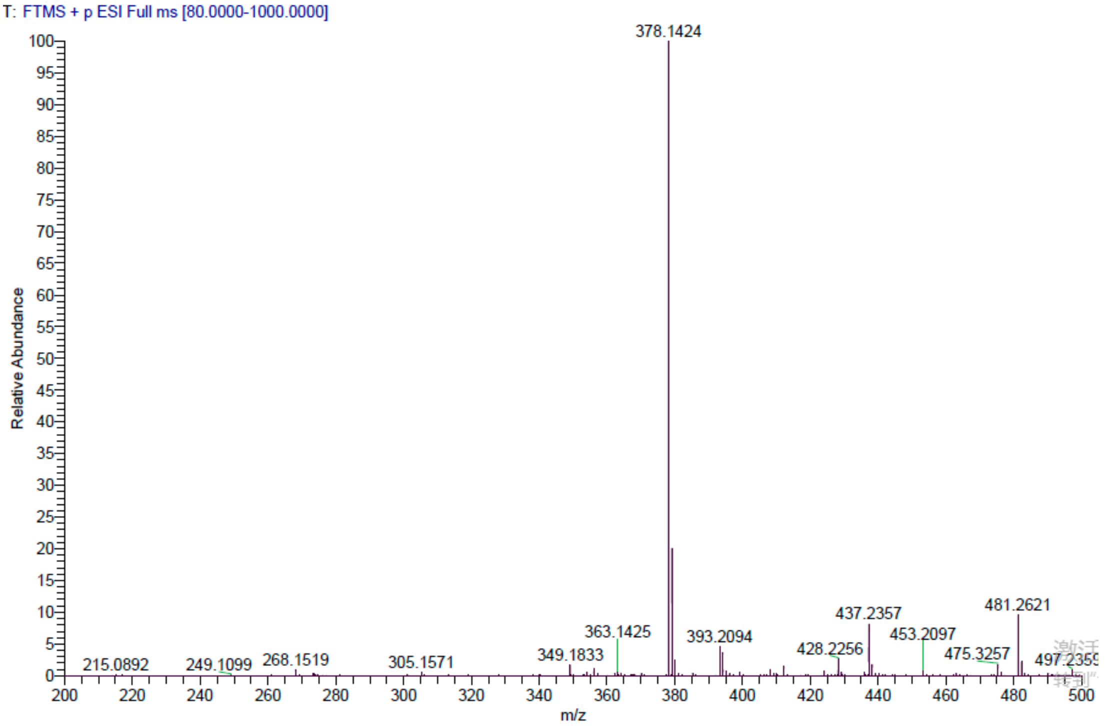

Figure S6. HRESIMS for compound **1**.

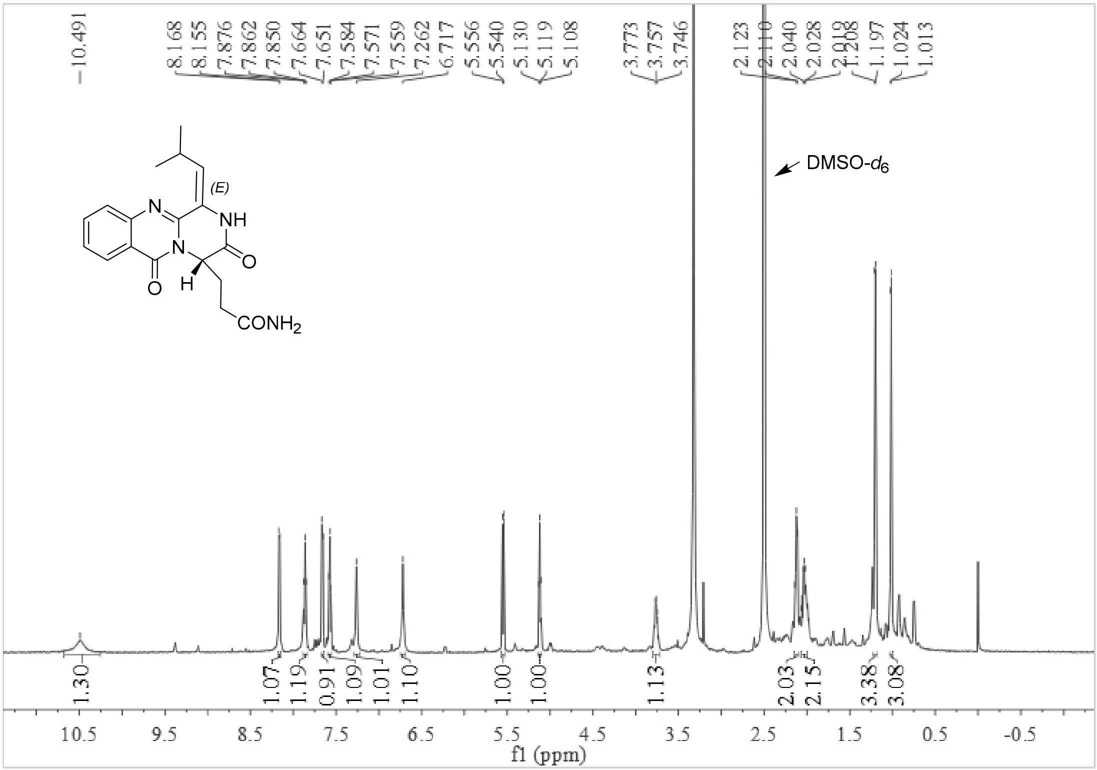

Figure S7. ^{1}H NMR (600 MHz, DMSO-d_6) spectrum of **2**

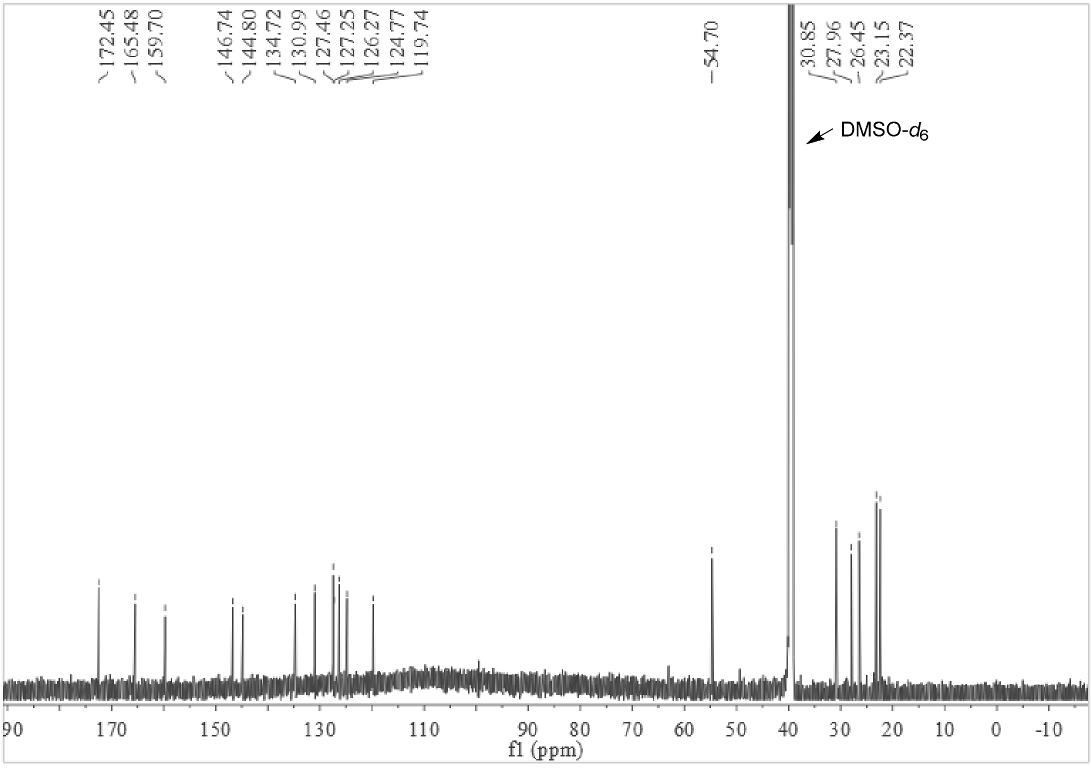

Figure S8. ^{13}C NMR (150 MHz, DMSO-d_6) spectrum of **2**.

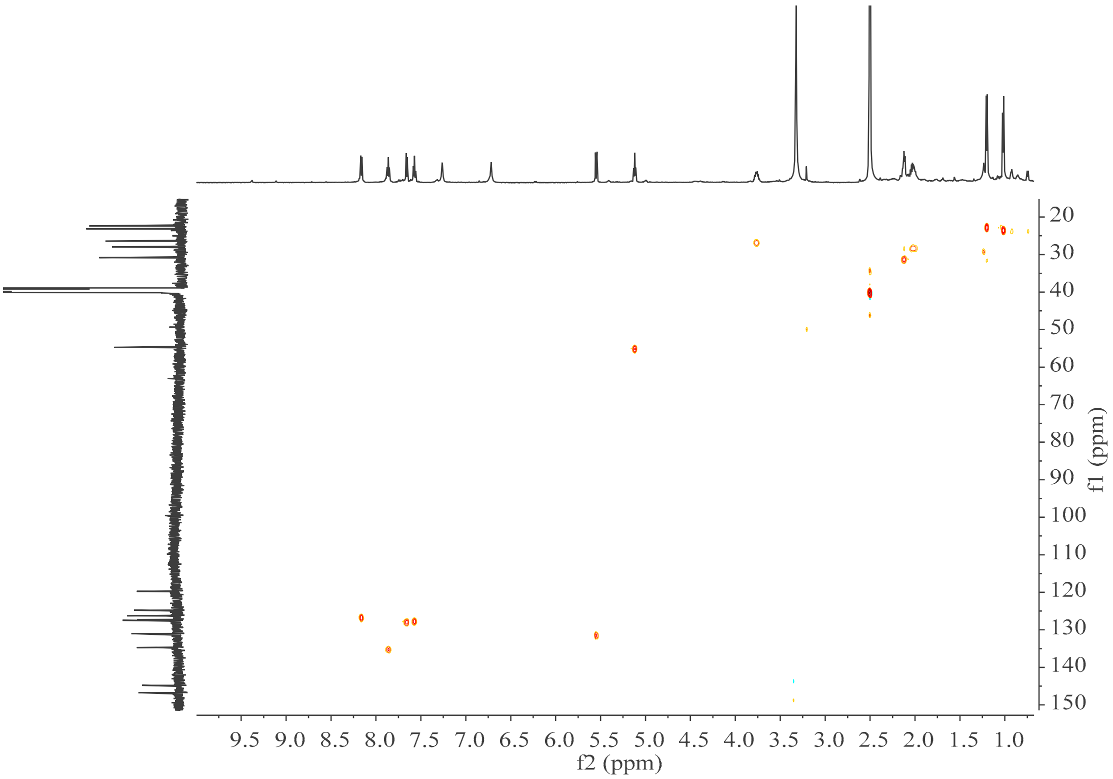

Figure S9. HSQC (DMSO-d_6) spectrum of **2.**

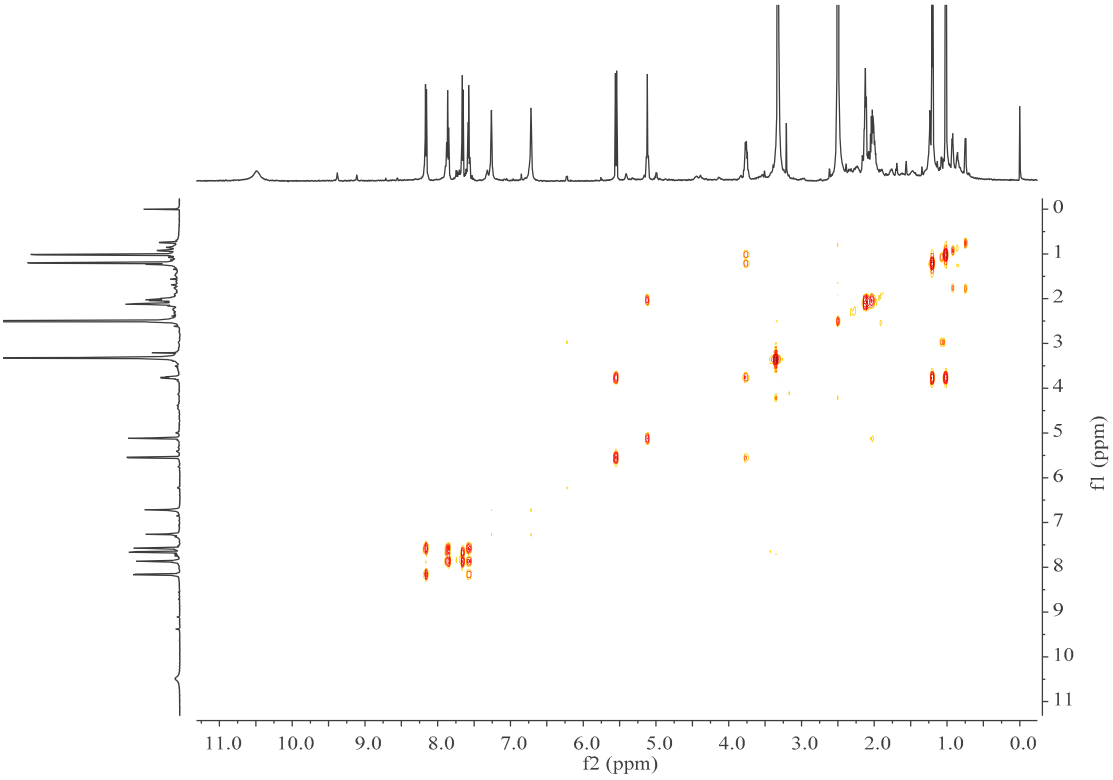

Figure S10. ^{1}H-^{1}H COSY (DMSO-d_6) spectrum of **2.**

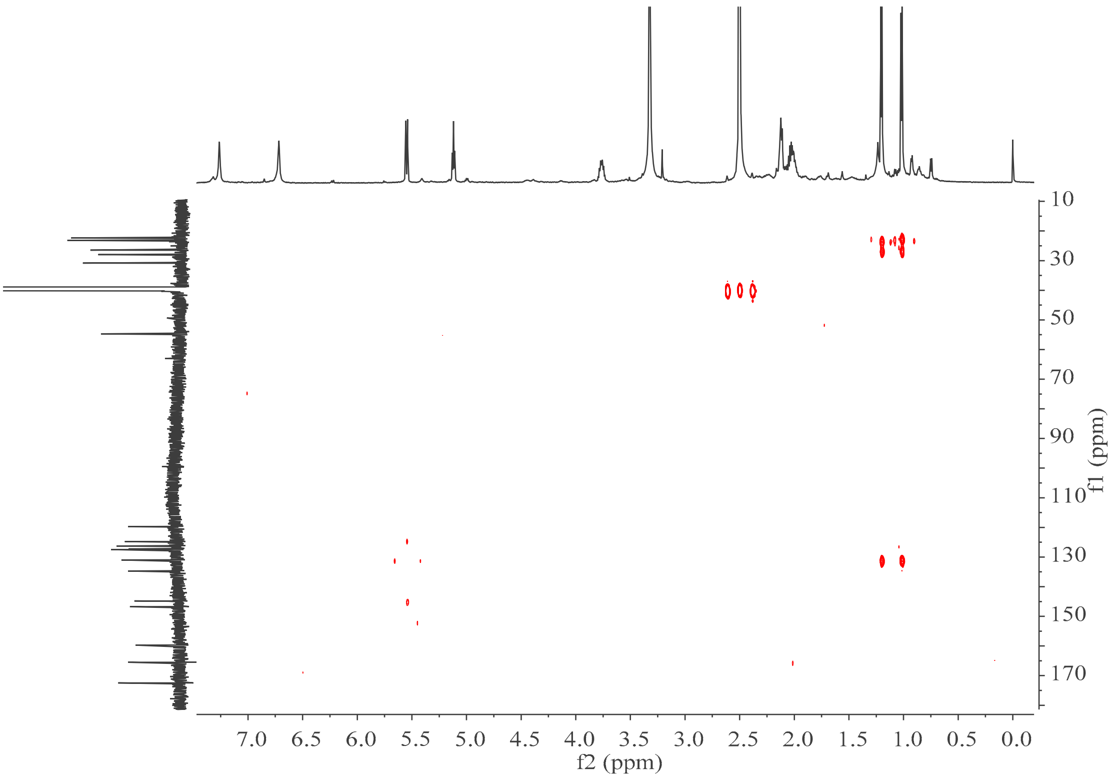

Figure S11. HMBC (DMSO-d_6) spectrum of **2.**

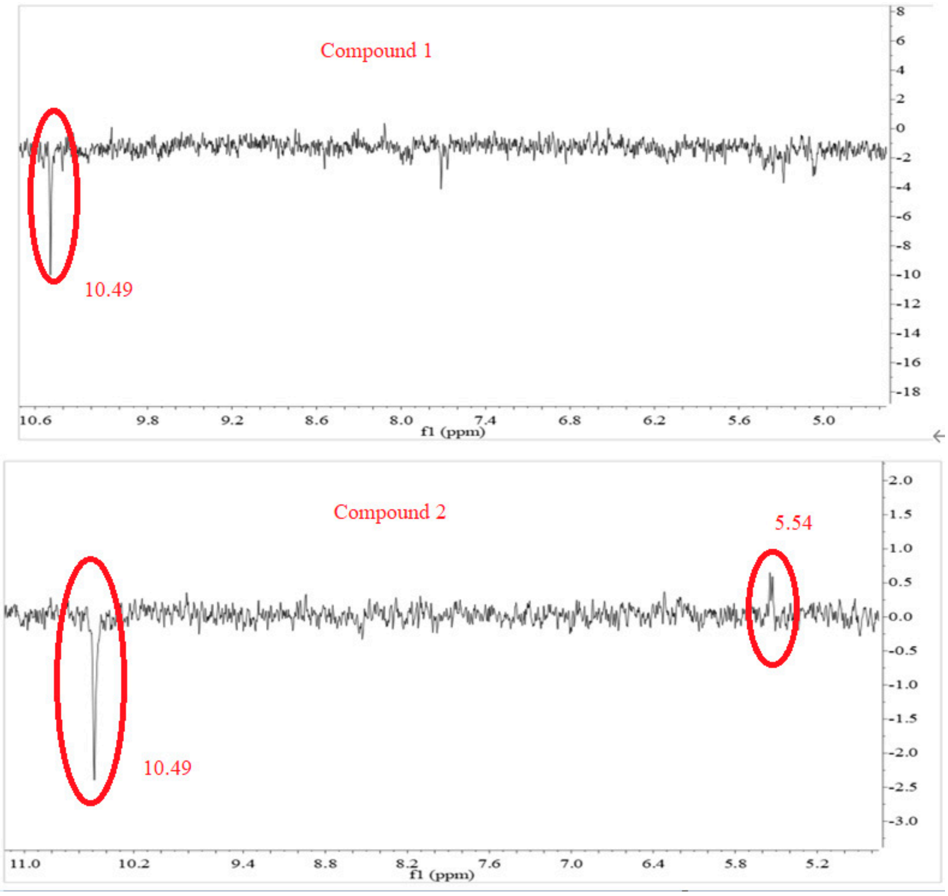

Figure S12. 1D NOE (DMSO-d_6) spectrum of compounds **1** and **2**.

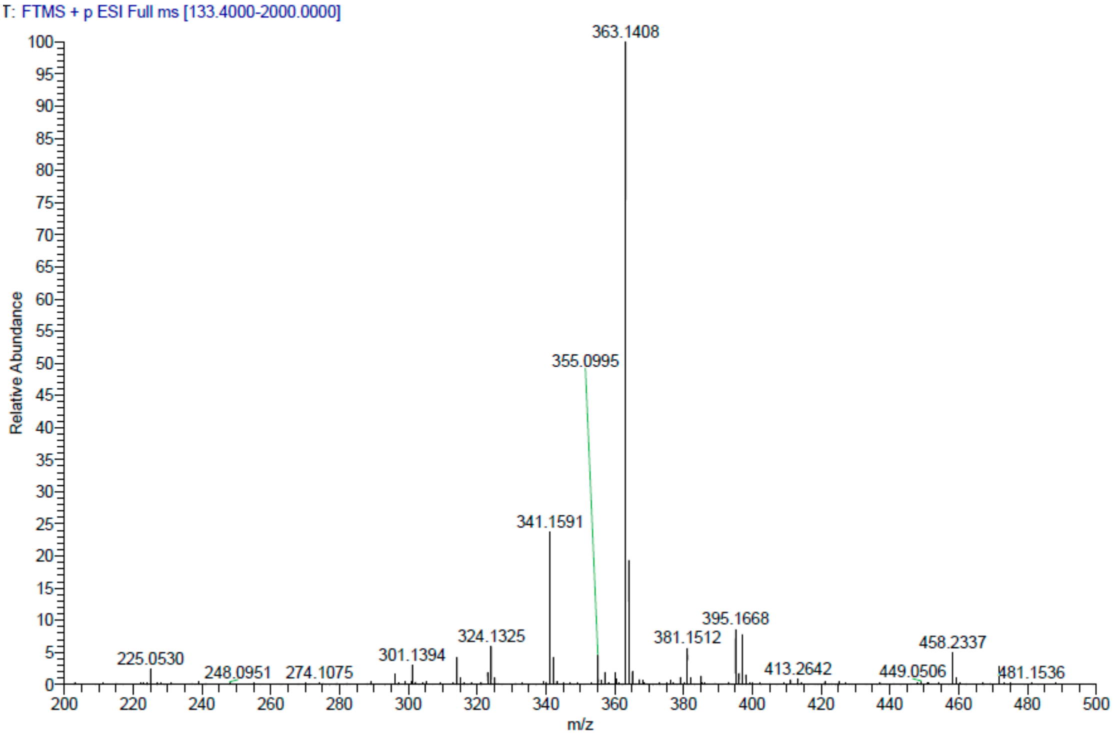

Figure S13. HRESIMS for compound **2.**

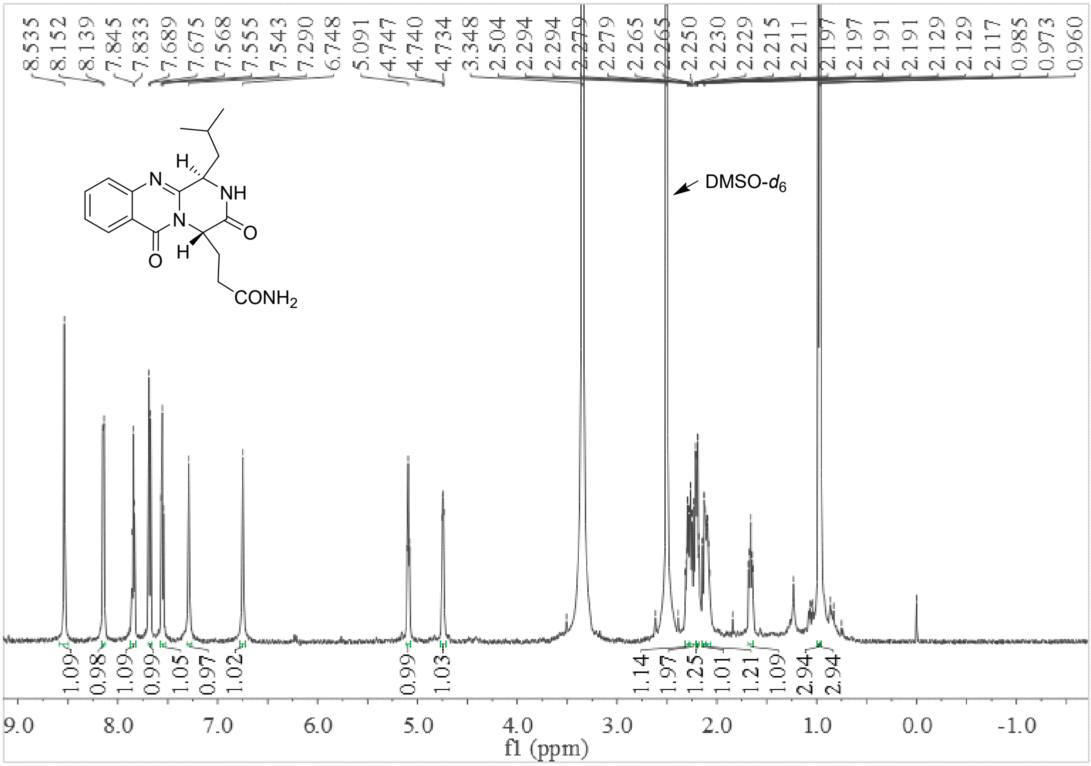

Figure S14. ^{1}H NMR (600 MHz, DMSO-d_6) spectrum of **3**.

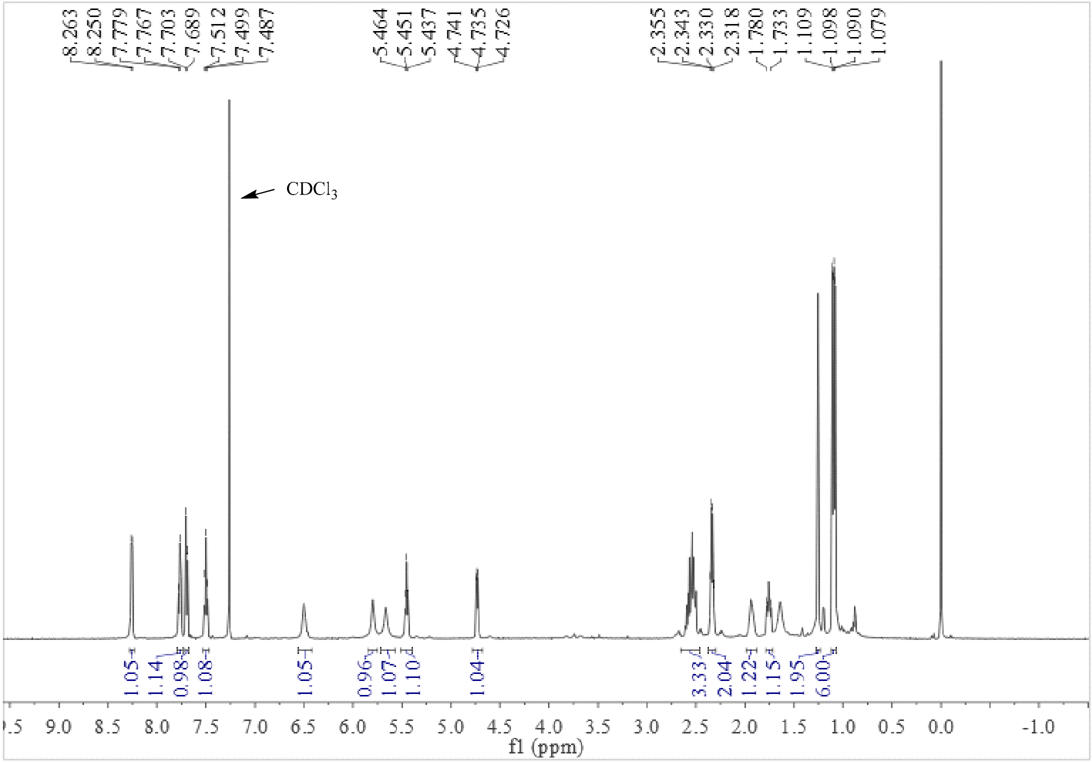

Figure S15. ^{1}H NMR (600 MHz, $CDCl_3$) spectrum of **3**.

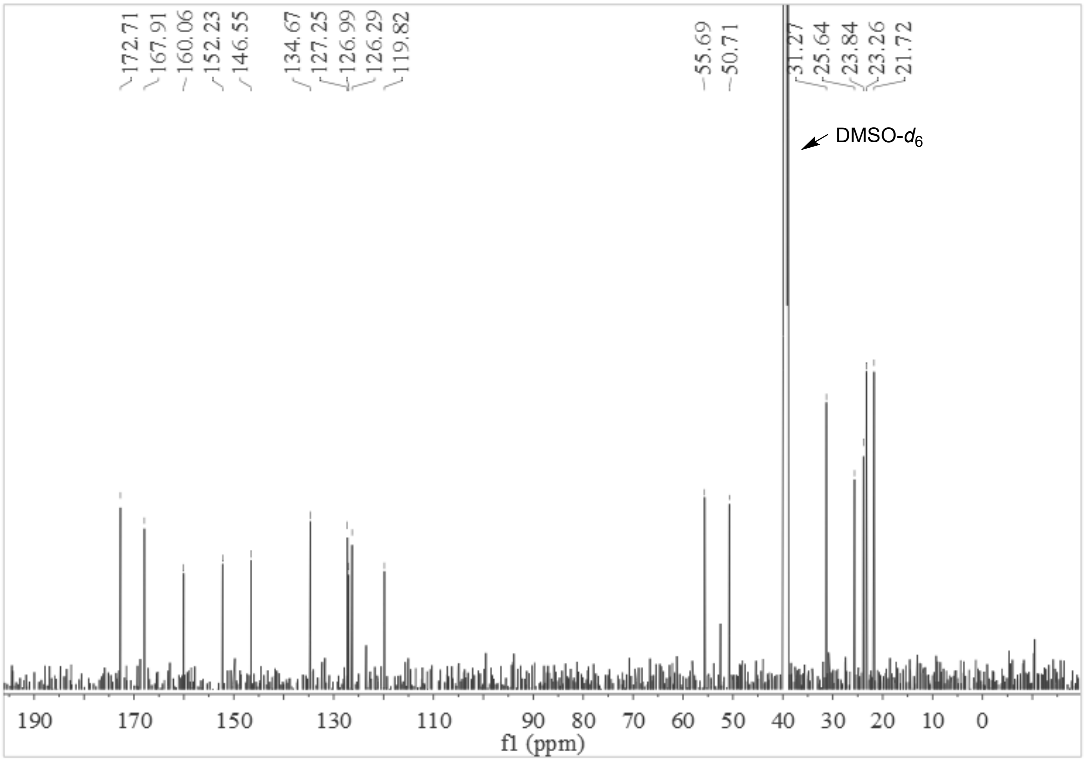

Figure S16. ^{13}C NMR (150 MHz, DMSO-d_6) spectrum of **3**.

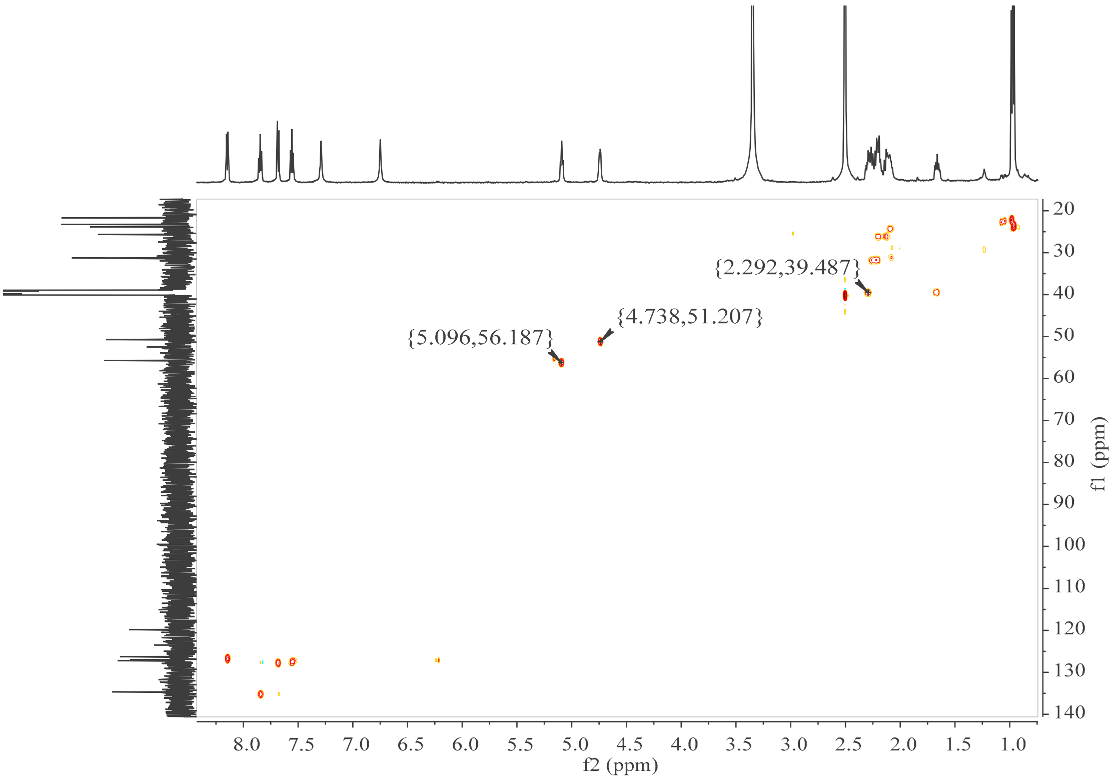

Figure S17. HSQC (DMSO-d_6) spectrum of **3.**

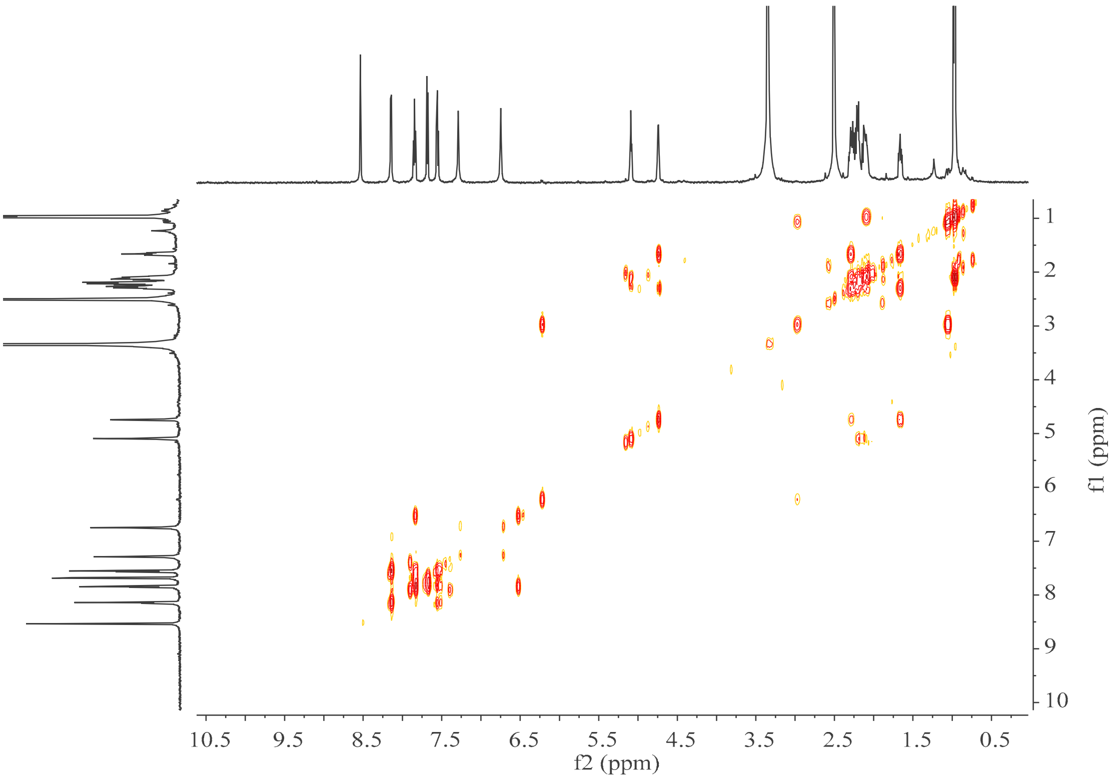

Figure S18. ^{1}H-^{1}H COSY (DMSO-d_6) spectrum of **3.**

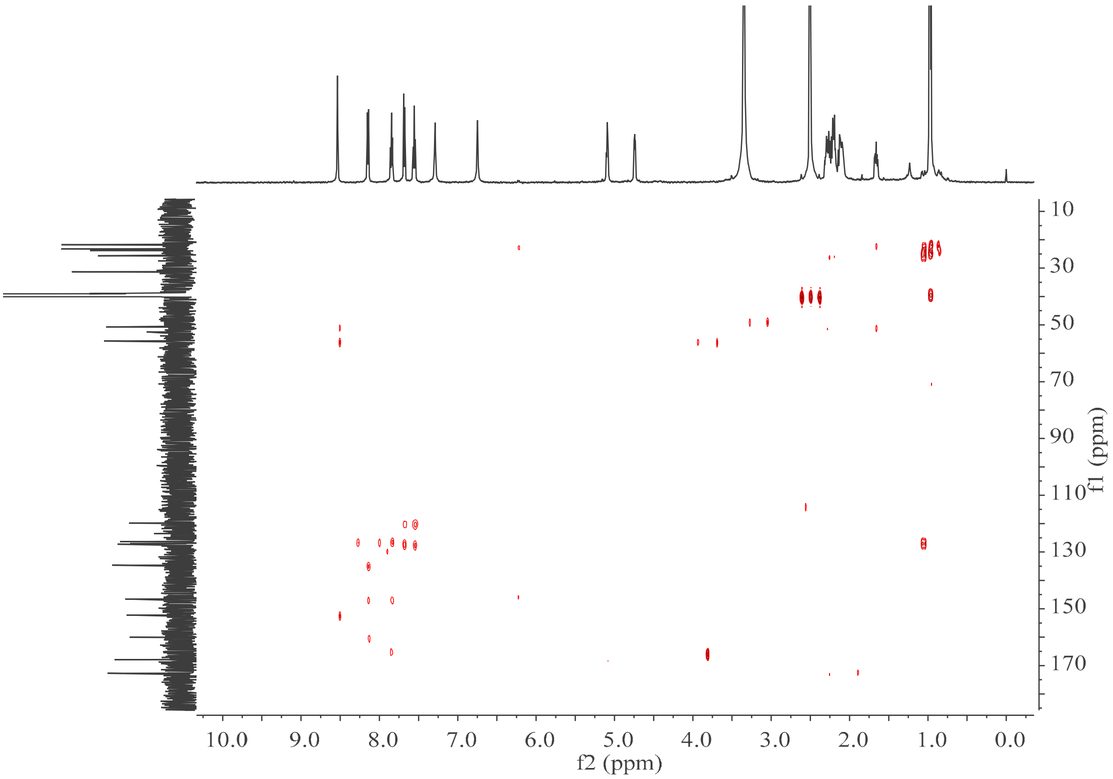

Figure S19. HMBC (DMSO-d_6) spectrum of **3.**

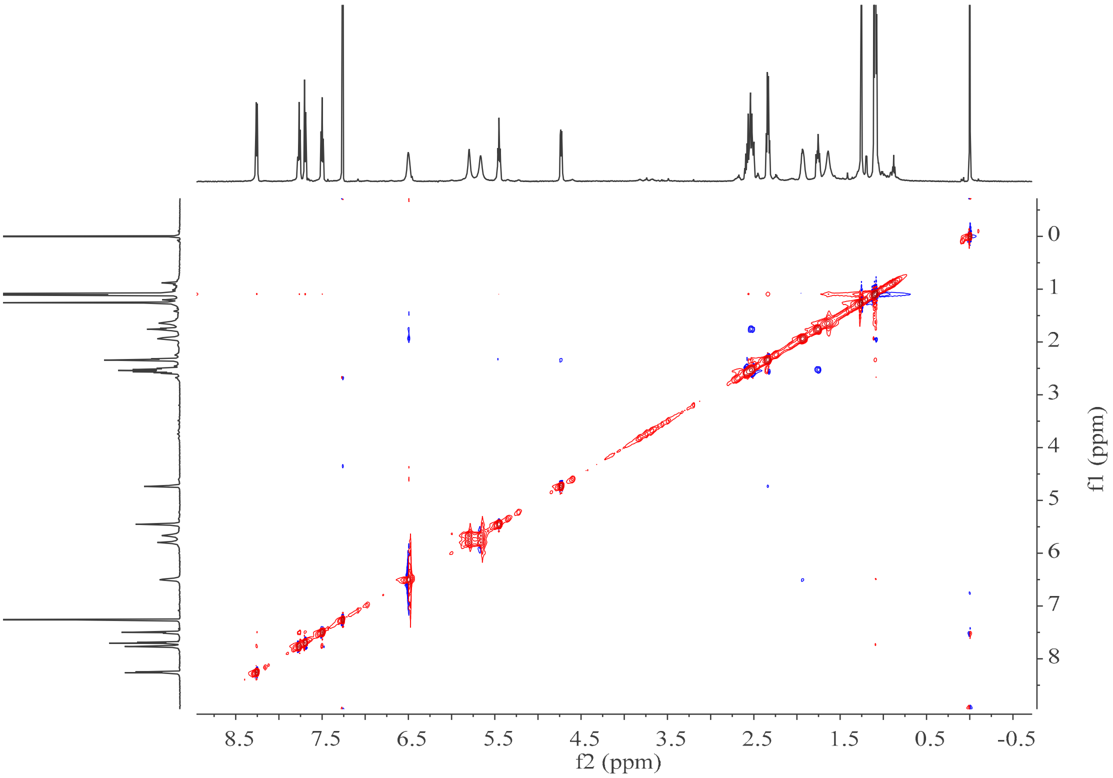

Figure S20. NOESY ($CDCl_3$) spectrum of compound **3.**

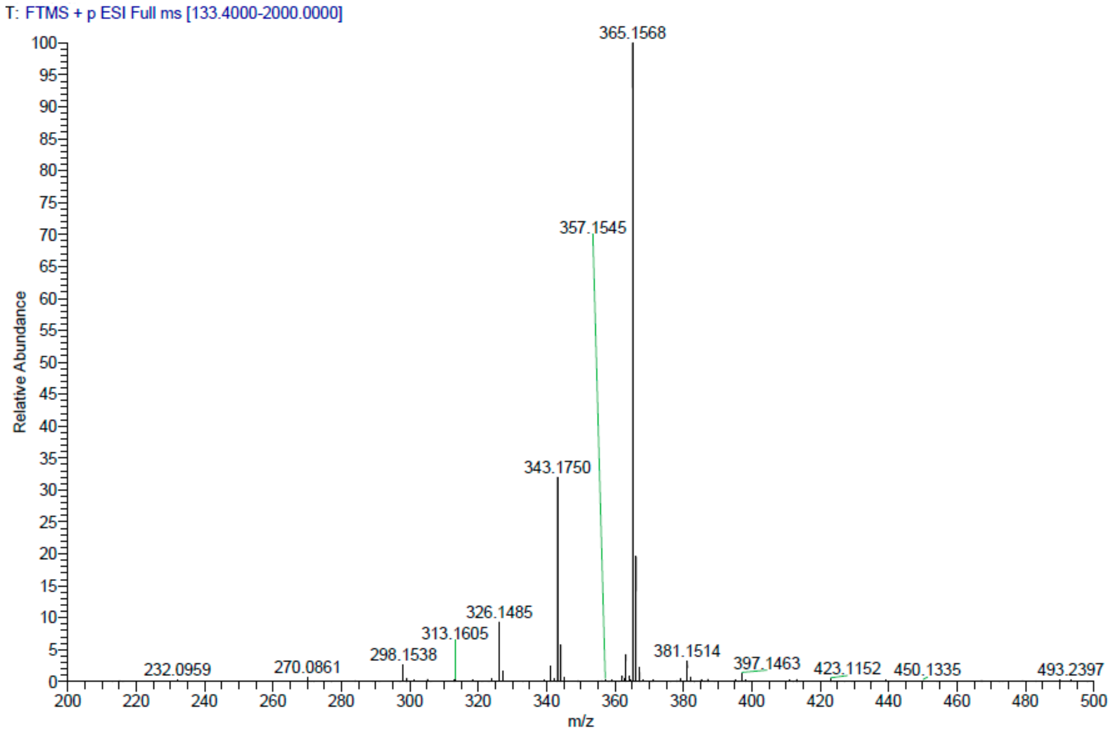

Figure S21. HRESIMS for compound **3.**

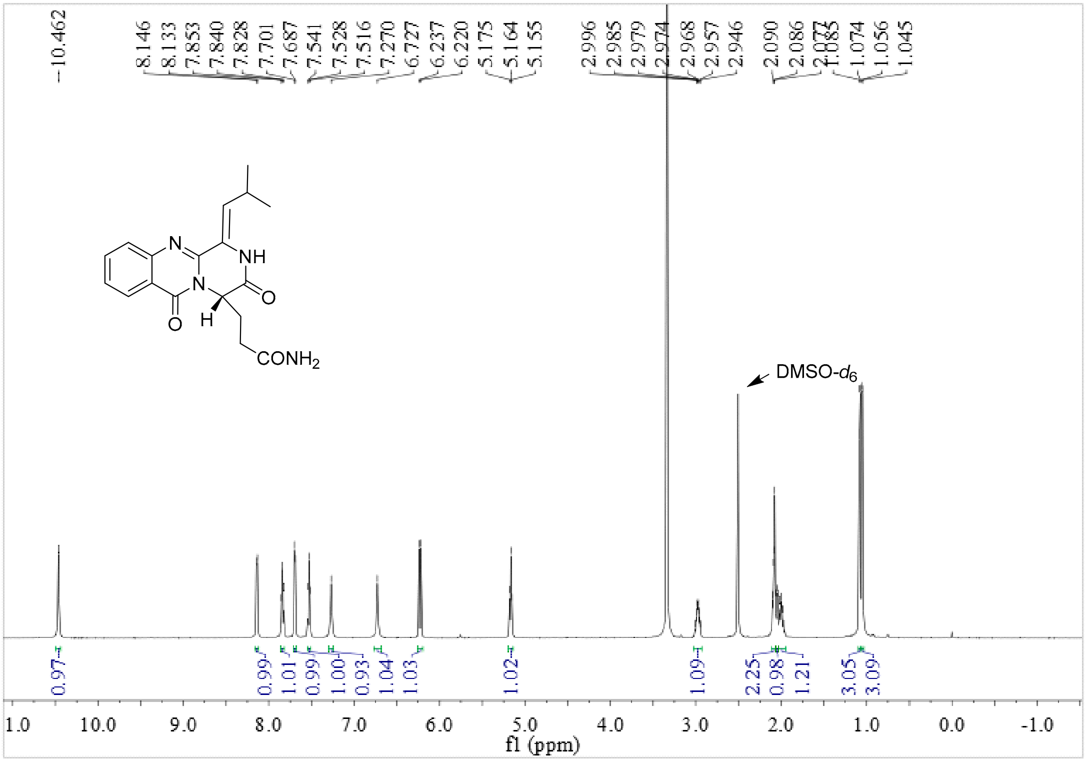

Figure S22. ^{1}H NMR (600 MHz, DMSO-*d*6) spectrum of **4**.

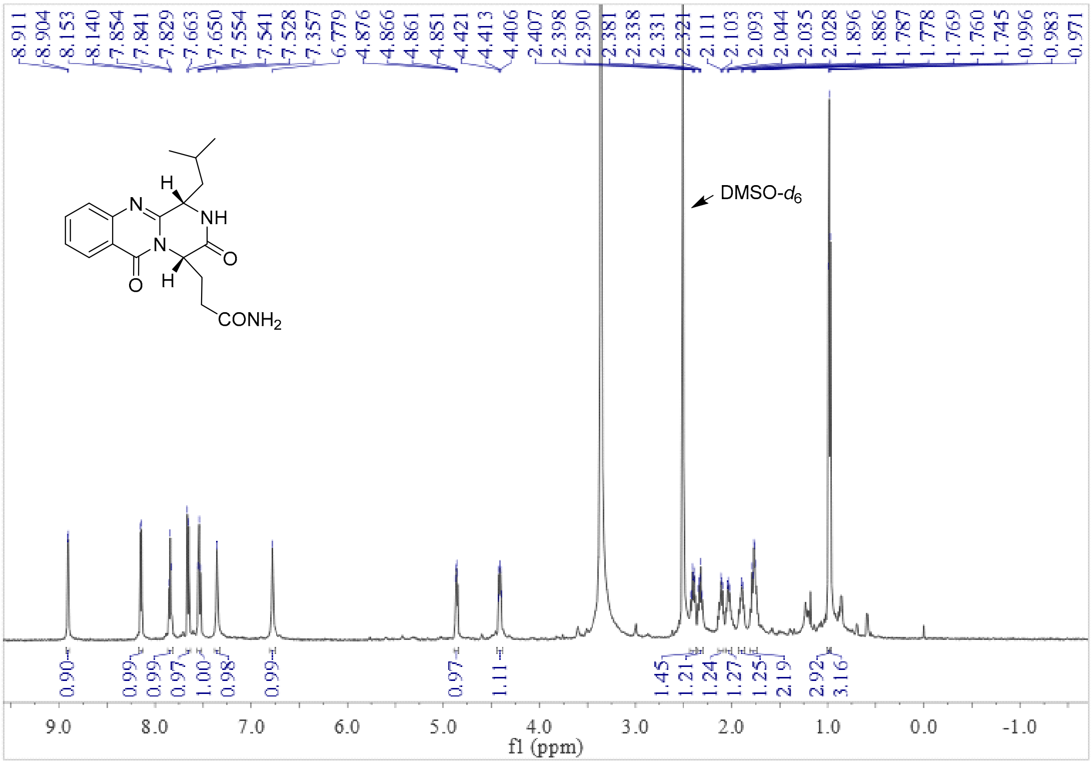

Figure S23. ^{1}H NMR (600 MHz, DMSO-*d*6) spectrum of **5**.

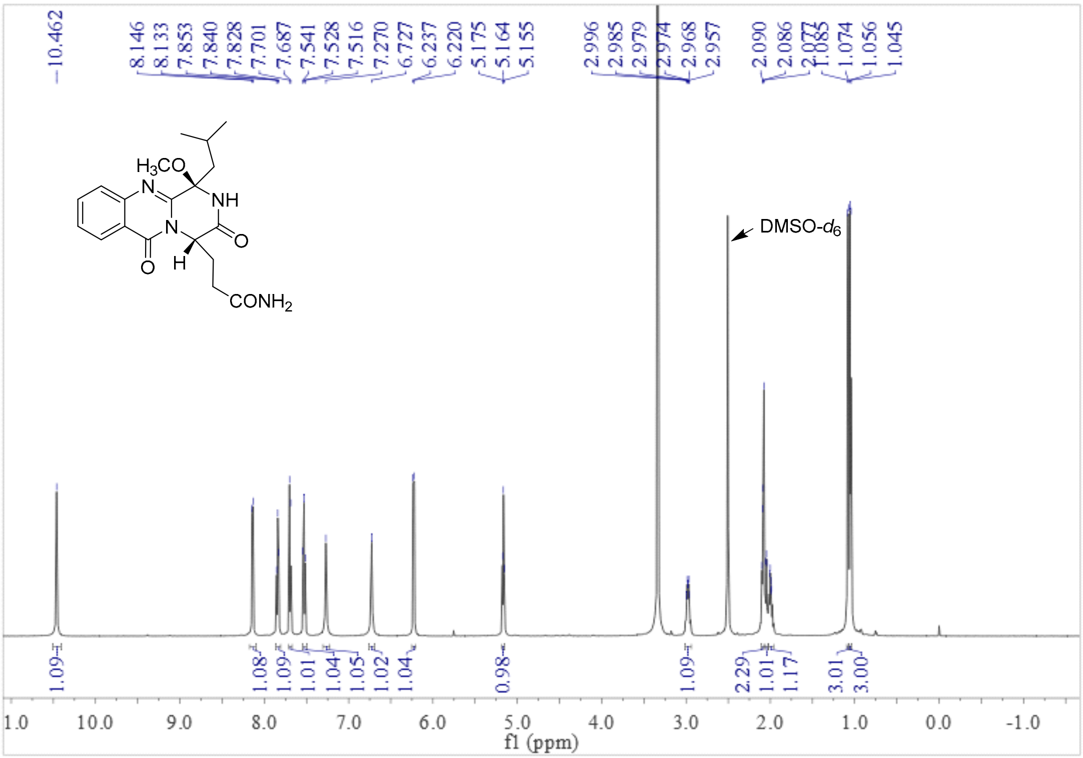

Figure S24. ^{1}H NMR (600 MHz, DMSO-*d*6) spectrum of **6**.

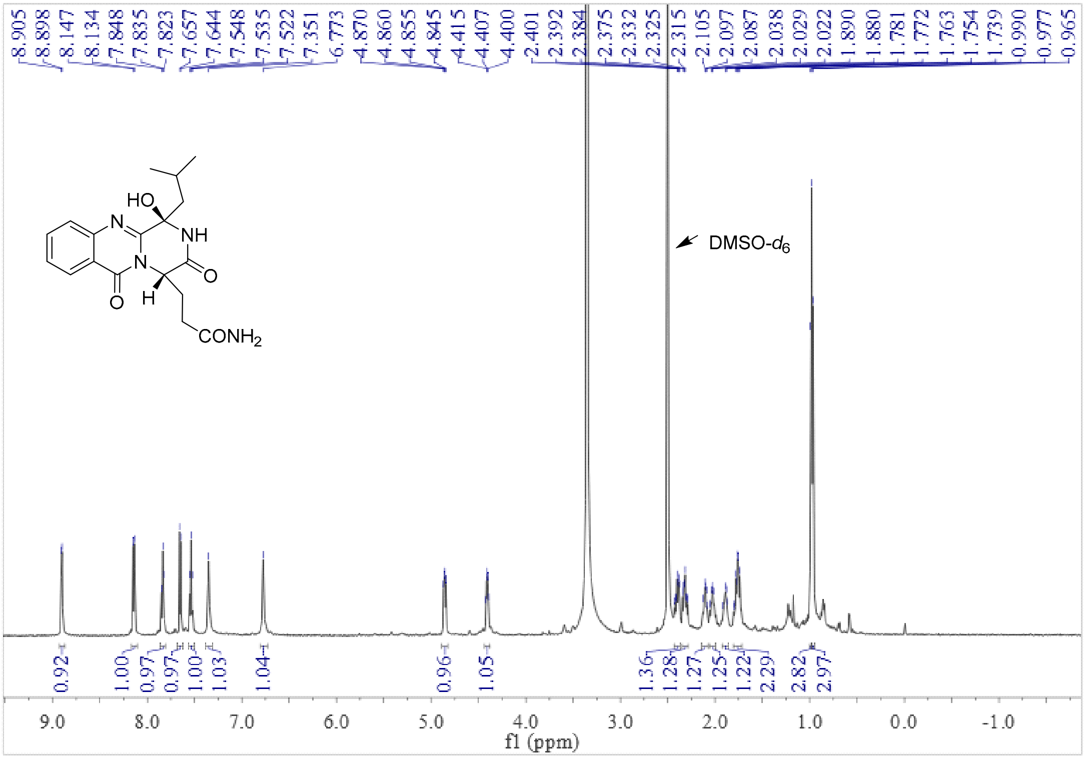

Figure S25. ^{1}H NMR (600 MHz, DMSO-d_6) spectrum of **7**.

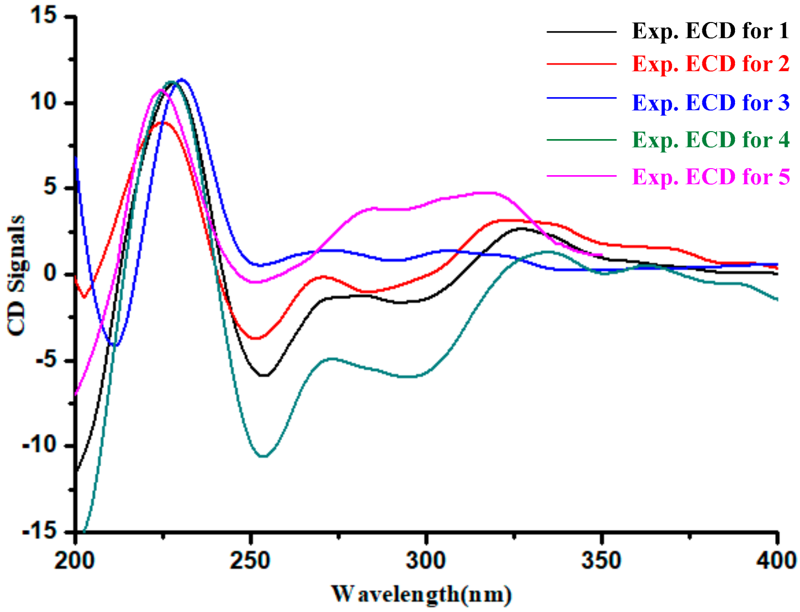

Figure S26. Experimental ECD spectra of **1**–**5**.

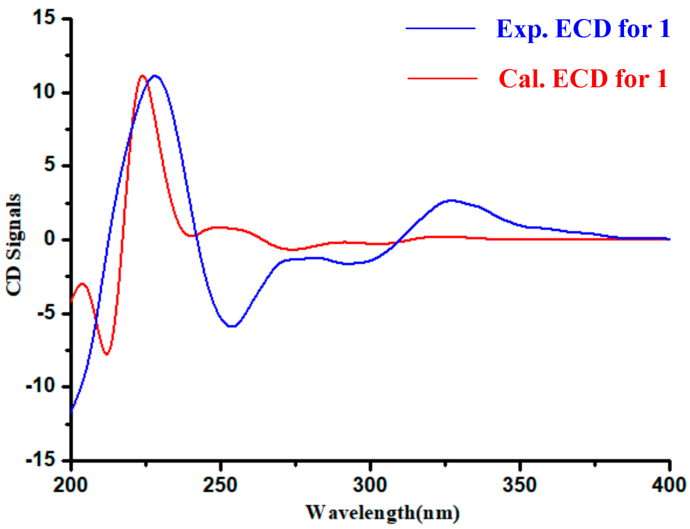

Figure S27. Experimental and calculated ECD spectra of **1**.

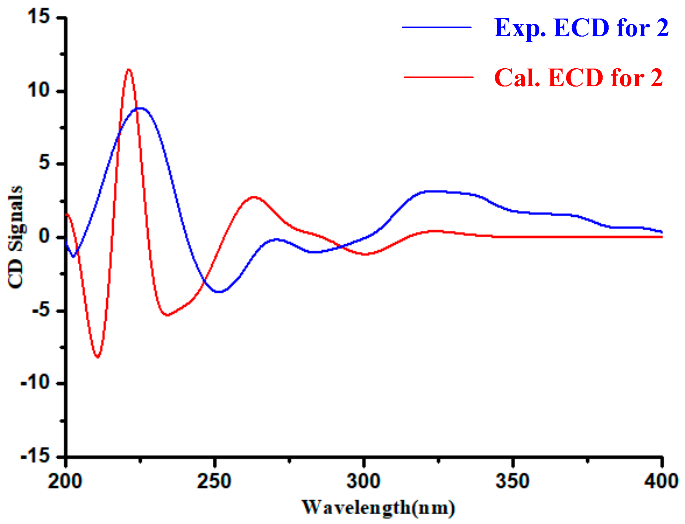

Figure S28. Experimental and calculated ECD spectra of **2**.

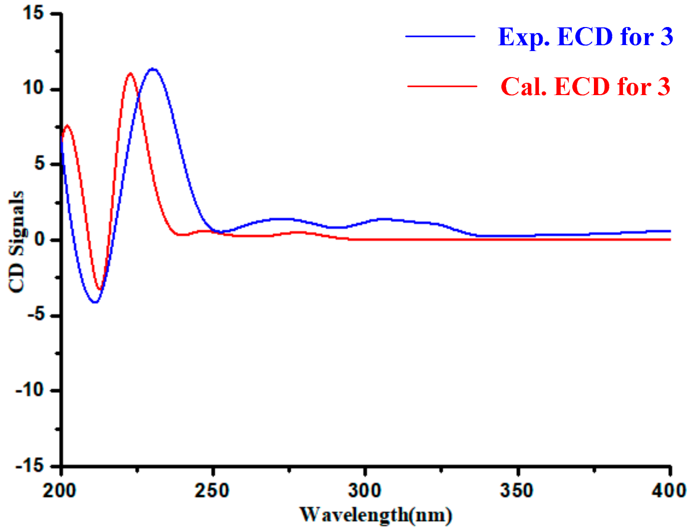

Figure S29. Experimental and calculated ECD spectra of **3**.

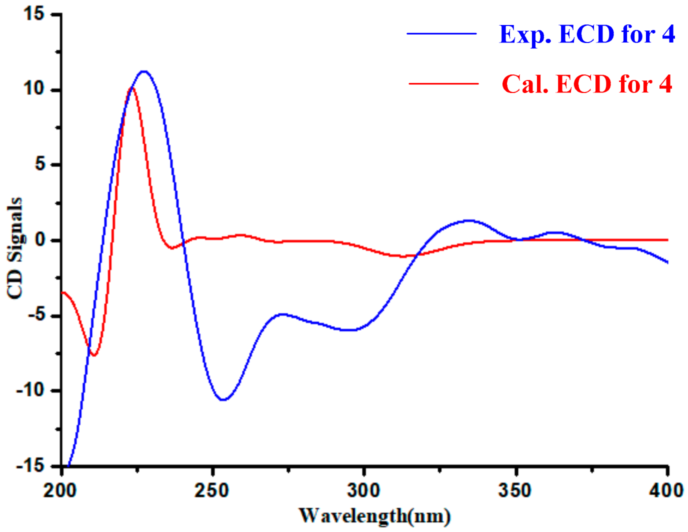

Figure S30. Experimental and calculated ECD spectra of **4**.

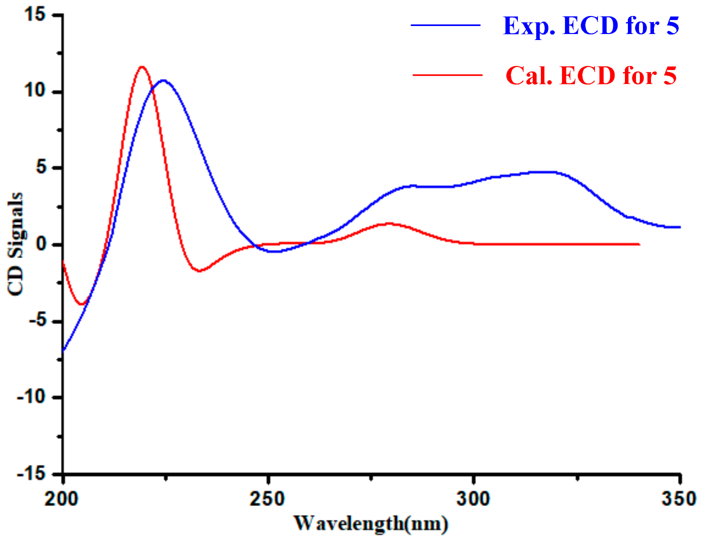

Figure S31. Experimental and calculated ECD spectra of **5**.

Functional		Solvent?		Basis Set	
B3LYP		Gas Phase		6-311+G(d,p)	
		DP4+	100.00%	0.00%	-
Nuclei	sp2?	Experimental	Isomer 1	Isomer 2	Isomer 3
C	x	134.7	134.2420294	134.3632431	
C	x	127	126.4808853	126.171998	
C	x	126.3	128.4799392	128.3022902	
C	x	119.8	121.2847373	120.6404755	
C	x	146.6	148.4599039	148.7865657	
C	x	127.3	128.5309245	128.0625951	
C	x	160.1	159.654048	159.6477137	
C	x	152.2	152.921151	152.6898206	
C		55.7	56.4478451	55.7540951	
C	x	167.9	167.820049	166.9695422	
C		50.7	51.55953235	57.17189902	
C		39	38.68519118	46.50892157	
C		23.8	27.0402049	26.99855392	
C		21.7	17.36431176	20.85926961	
C		23.3	22.18061765	18.03609902	
C		25.6	26.0581402	29.29893529	
C		31.3	31.09409118	31.59072451	
C	x	172.7	171.0076245	171.0901745	
H		5.09	4.479535981	3.94216221	
H	x	7.68	7.385941048	7.283663257	
H	x	7.85	7.386634648	7.247590695	
H	x	7.56	7.133378952	6.986158467	
H	x	8.15	8.125107962	7.984918019	
H		4.74	5.040505524	4.7519782	
H		2.2	2.011418514	2.0901142	
H		2.24	2.109524038	2.035519295	
H		2.29	2.237236648	1.772153333	
H		1.66	1.432108762	1.504773133	
H		0.98	0.998012781	0.736730771	
H		0.97	0.967706933	0.833866333	

Figure S32. The data of DP4plus method of compound **3** (B3lyp/6-311+G(d,p))

Functional		Solvent?		Basis Set	
B3LYP		Gas Phase		6-311+G(d,p)	
		DP4+	0.00%	100.00%	-
Nuclei	sp2?	Experimental	Isomer 1	Isomer 2	Isomer 3
C	x	134.7	134.2420294	134.3632431	
C	x	126.7	126.4808853	126.171998	
C	x	126.2	128.4799392	128.3022902	
C	x	119.7	121.2847373	120.6404755	
C	x	147	148.4599039	148.7865657	
C	x	126.7	128.5309245	128.0625951	
C	x	160.1	159.654048	159.6477137	
C	x	152	152.921151	152.6898206	
C		54.9	56.4478451	55.7540951	
C	x	166.6	167.820049	166.9695422	
C		53.8	51.55953235	57.17189902	
C		47.2	38.68519118	46.50892157	
C		24	27.0402049	26.99855392	
C		21.4	17.36431176	20.85926961	
C		23	22.18061765	18.03609902	
C		29.4	26.0581402	29.29893529	
C		32.2	31.09409118	31.59072451	
C	x	172.8	171.0076245	171.0901745	
H		4.41	4.479535981	3.94216221	
H	x	7.66	7.385941048	7.283663257	
H	x	7.84	7.386634648	7.247590695	
H	x	7.54	7.133378952	6.986158467	
H	x	8.15	8.125107962	7.984918019	
H		4.86	5.040505524	4.7519782	
H		2.06	2.011418514	2.0901142	
H		2.35	2.109524038	2.035519295	
H		1.76	1.50710861	1.772153333	
H		1.9	1.432108762	1.504773133	
H		0.97	0.998012781	0.736730771	
H		0.99	0.967706933	0.833866333	

Figure S33. The data of DP4plus method of compound **5** (B3lyp/6-311+G(d,p))

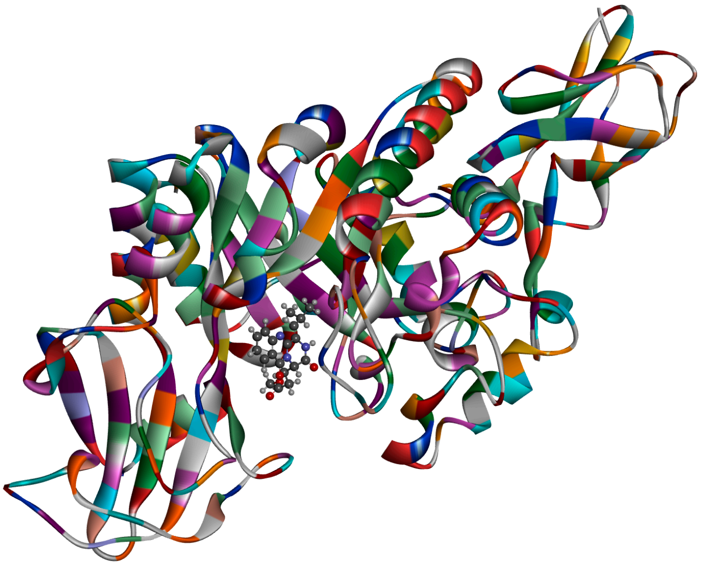

Figure S34. Compound **1** tightly bind to the entire active pocket of *Of*Chi-h

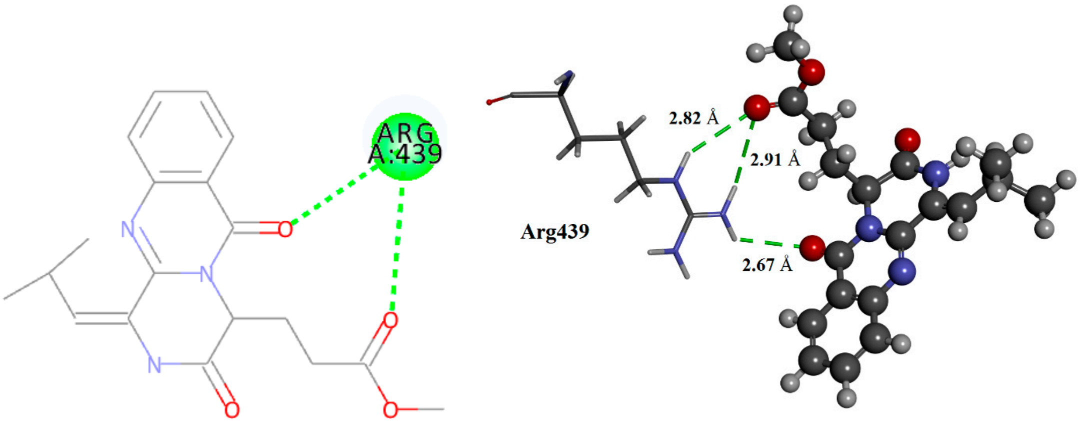

Figure S35. Three hydrogen bonds formed by **1** with the guanidine group of ARG439

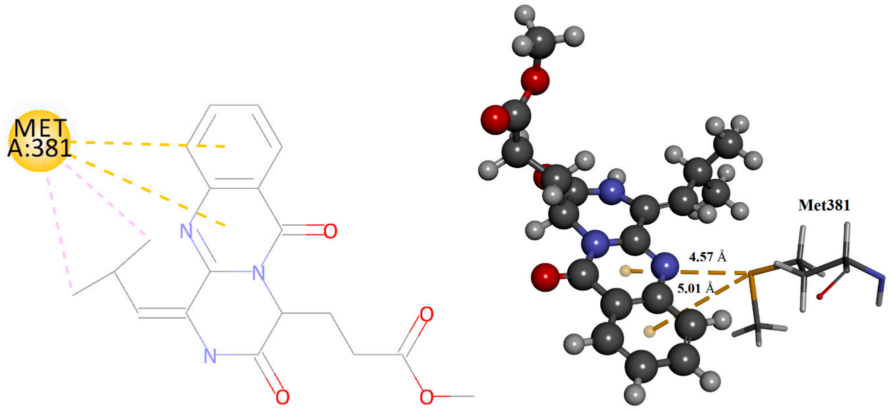

Figure S36. π-Sulfur interaction between **1** and the sulfur atom of methionine MET381

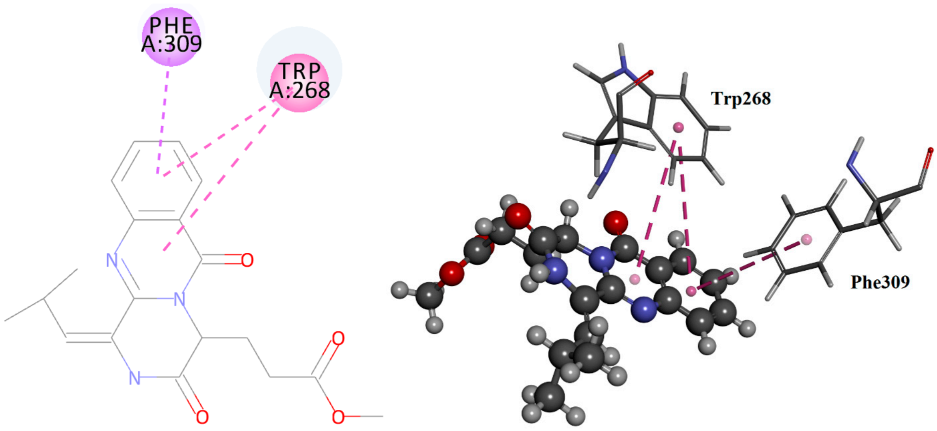

Figure S37. π-π Stacking between **1** and the benzene rings of Trp268 and Phe309

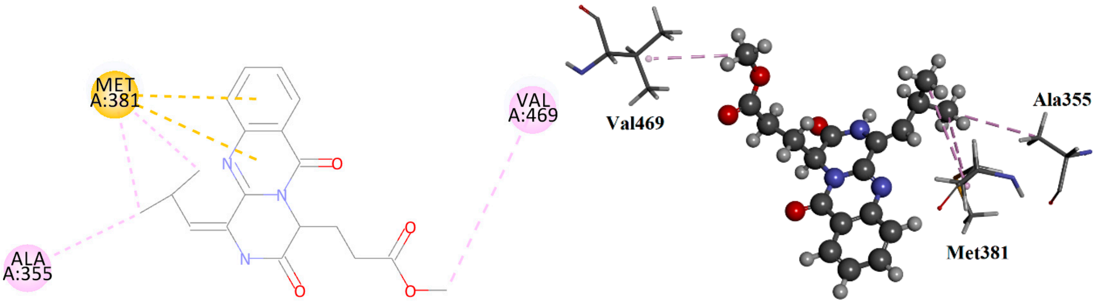

Figure S38. Alkyl hydrophobic interactions between **1** and Ala355/Met381, and between **1** and Val469

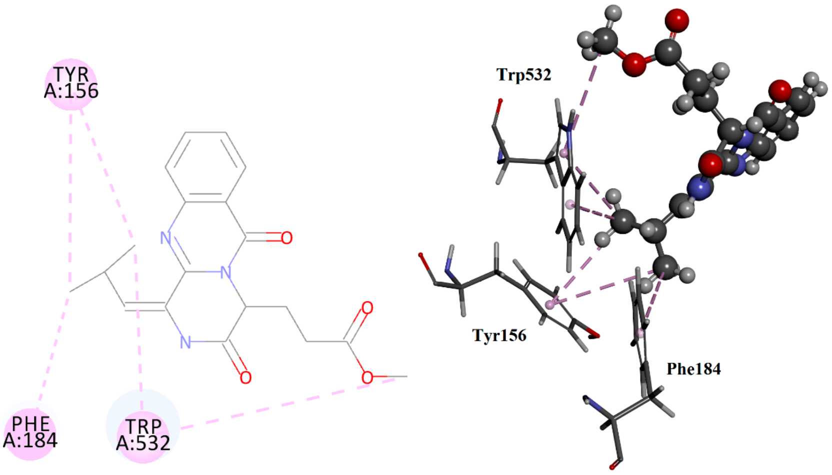

Figure S39. Mixed π/alkyl hydrophobic interactions between **1** and Tyr156, and between **1** and Phe184

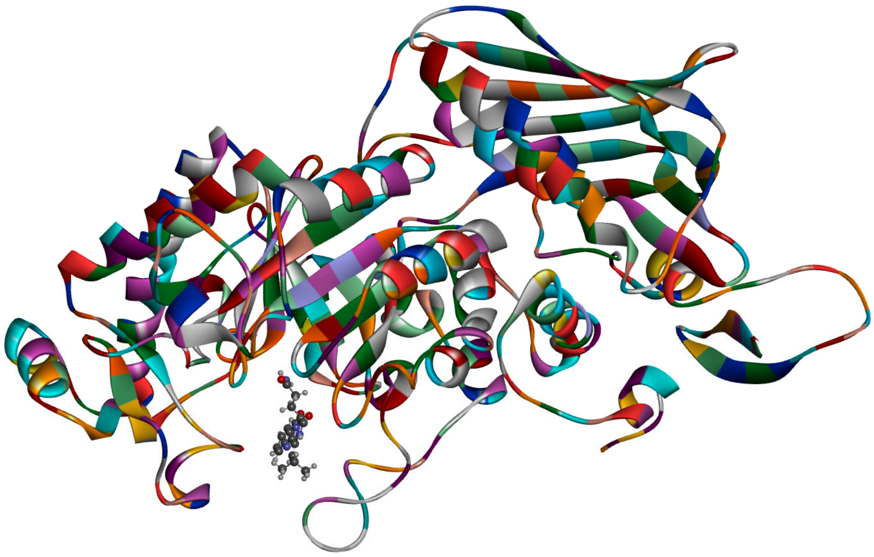

Figure S40. Compound **4** tightly bind to the *Of*Hex1 in a "U" conformation

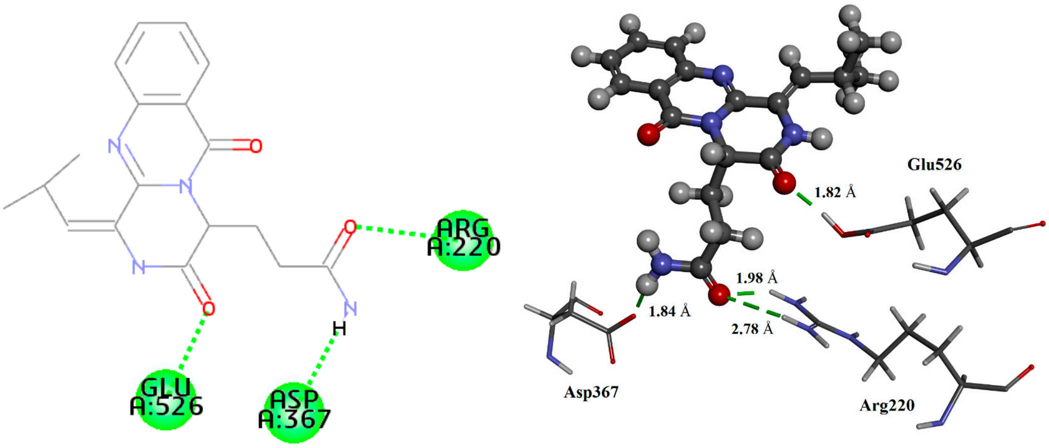

Figure S41. Hydrogen bonds formed between **4** and the guanidine group of ARG220, and between **4** and the carboxyl of ASP367

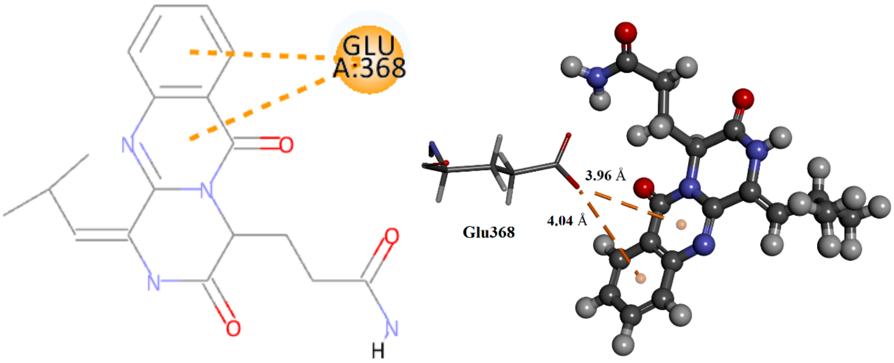

Figure S42. Compound **4** had a π-anion with the carboxyhydroxyl oxygen anion in the residue of GLU368

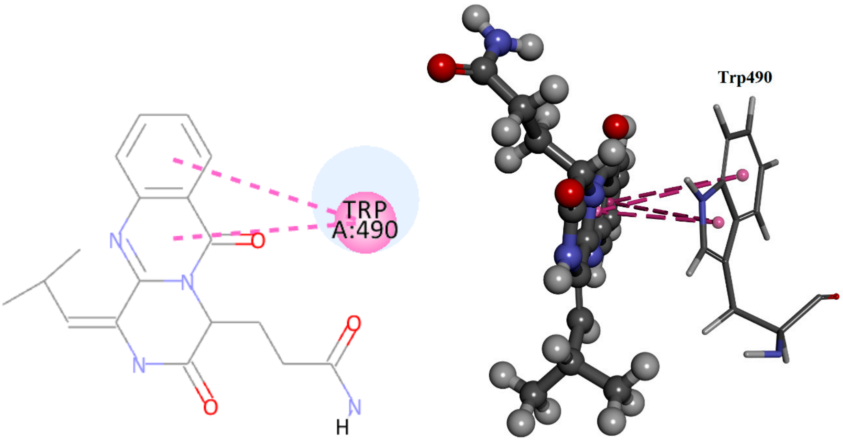

Figure S43. π-π Stacking interaction between **4** and the indole ring of Trp490

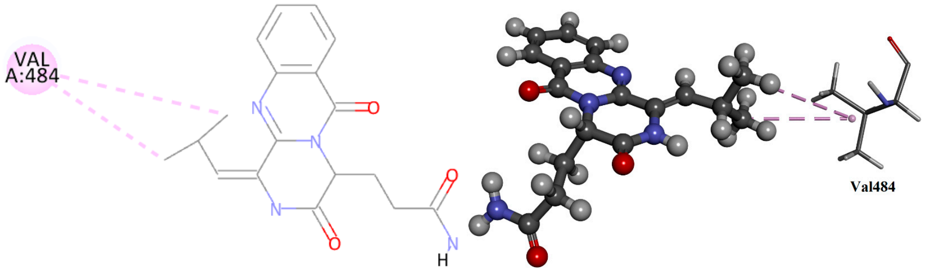

Figure S44. Alkyl hydrophobic interaction between **4** and the isopropyl group of Val484

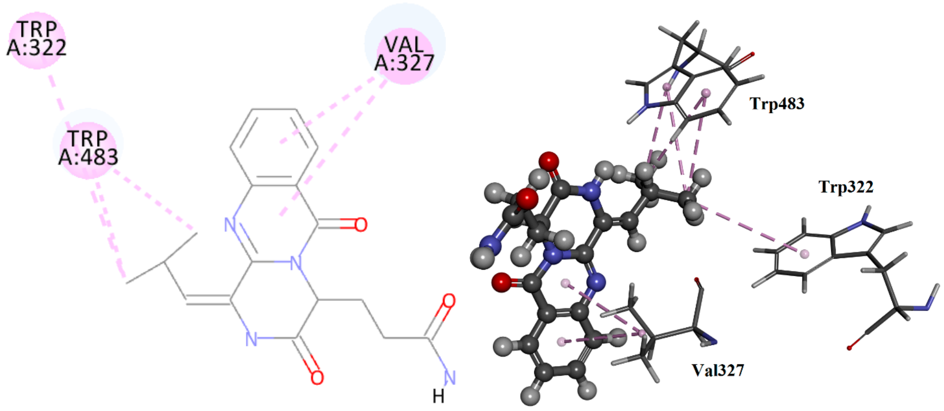

Figure S45. Mixed π/alkyl hydrophobic interactions between **4** and Trp322/Trp483, and between **4** and the isopropyl group of Val327

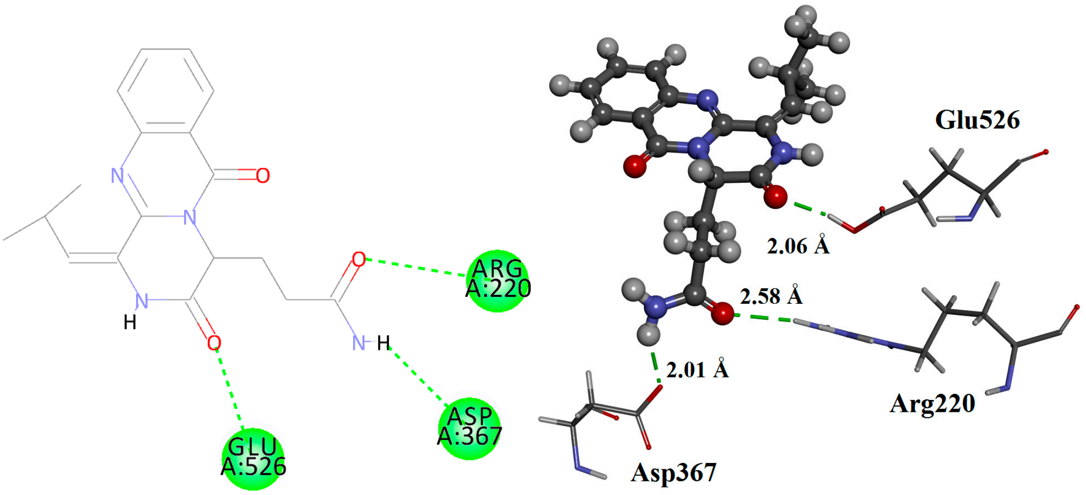

Figure S46. N-H hydrogen bond interaction between **2** and Arg220

Table S1. The data of DP4plus method of compound **3** and **5**

Nuclei	3-Exp.	3-Cal.3*R*,14*S*	5-Exp.	5-Cal.3*S*,14*S*
C-8	134.7	134.242	134.7	134.3632
C-9	127	126.4809	126.7	126.172
C-10	126.3	128.4799	126.2	128.3023
C-11	119.8	121.2847	119.7	120.6405
C-6	146.6	148.4599	147	148.7866
C-7	127.3	128.5309	126.7	128.0626
C-12	160.1	159.654	160.1	159.6477
C-4	152.2	152.9212	152	152.6898
C-3	55.7	56.44785	54.9	55.7541
C-1	167.9	167.82	166.6	166.9695
C-14	50.7	51.55953	53.8	57.1719
C-18	39	38.68519	47.2	46.50892
C-19	23.8	27.0402	24	26.99855
C-21	21.7	17.36431	21.4	20.85927
C-20	23.3	22.18062	23	18.0361
C-15	25.6	26.05814	29.4	29.29894
C-16	31.3	31.09409	32.2	31.59072
C-17	172.7	171.0076	172.8	171.0902
H-3	4.74	4.479536	4.41	3.942162
H-7	7.68	7.385941	7.66	7.283663
H-8	7.85	7.386635	7.84	7.247591
H-9	7.56	7.133379	7.54	6.986158
H-10	8.15	8.125108	8.15	7.984918
H-14	5.09	5.040506	4.86	4.751978
H-15	2.18	2.011419	2.06	2.090114
H-16	2.24	2.109524	2.35	2.035519
H-18	2.29	2.237237	1.76	1.772153
H-19	2.1	1.432109	1.9	1.504773
H-21	0.98	0.998013	0.97	0.736731
H-20	0.97	0.967707	0.99	0.833866

Table S2. Cytotoxic activity data of compounds **1–7**

Compd.	IC_{50} (μM)		
	A549	HGC-27	UMUC-3
**1**	>10	>10	>10
**2**	>10	>10	>10
**3**	>10	>10	>10
**4**	>10	>10	>10
**5**	6.0	6.2	7.2
**6**	>10	>10	>10
**7**	>10	>10	>10
**DDP**	3.1	0.8	1.8

Table S3. The coordinate for the lowest-energy conformer of compound **1** for ECD calculation.

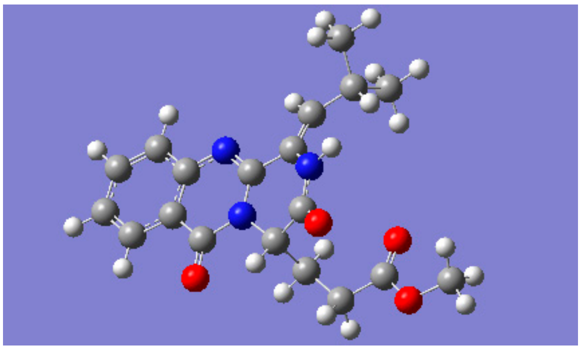

E=-1202.2385103 a.u.

Standard orientation:

Center Number	Atomic Number	Atomic Type	Coordinates (Angstroms) X	Y	Z
1	6	0	-5.712213	0.122134	-0.606088
2	6	0	-5.645965	-1.266240	-0.390490
3	6	0	-4.436573	-1.857697	-0.073716
4	6	0	-3.279302	-1.070387	0.034140
5	6	0	-3.339313	0.323463	-0.177906
6	6	0	-4.580173	0.908676	-0.501551
7	6	0	-1.992437	-1.690273	0.337346
8	7	0	-0.913627	-0.776280	0.412868
9	6	0	-1.092404	0.584867	0.225586
10	7	0	-2.230805	1.133145	-0.058420
11	6	0	0.430214	-1.369961	0.579110
12	6	0	1.376786	-0.445246	1.325816
13	7	0	1.143582	0.887338	1.165696
14	6	0	0.103603	1.447800	0.397243
15	8	0	-1.803560	-2.885775	0.498808
16	6	0	0.140339	2.693743	-0.101104
17	8	0	2.304341	-0.881035	1.985534
18	1	0	0.299749	-2.256852	1.196618
19	6	0	0.992491	-1.801797	-0.798481
20	6	0	2.357860	-2.492331	-0.743409
21	6	0	1.264237	3.687003	-0.010664
22	6	0	1.688219	4.134896	-1.421630
23	6	0	0.847324	4.896934	0.848280
24	6	0	3.534107	-1.535705	-0.722251
25	8	0	3.489333	-0.363455	-1.014620
26	8	0	4.666246	-2.175304	-0.380948
27	6	0	5.858514	-1.373333	-0.327721

28	1	0	-6.663612	0.581403	-0.855196
29	1	0	-6.542868	-1.870545	-0.473671
30	1	0	-4.354487	-2.925381	0.093623
31	1	0	-4.617457	1.980625	-0.659409
32	1	0	1.847612	1.498563	1.555254
33	1	0	-0.758103	3.021264	-0.614381
34	1	0	0.261805	-2.493688	-1.221831
35	1	0	1.047143	-0.932914	-1.459399
36	1	0	2.441364	-3.173181	0.106446
37	1	0	2.483664	-3.114469	-1.637068
38	1	0	2.141522	3.224928	0.457642
39	1	0	2.502744	4.863072	-1.366713
40	1	0	2.030246	3.288062	-2.020747
41	1	0	0.853907	4.607786	-1.949472
42	1	0	1.663579	5.622213	0.916108
43	1	0	0.574508	4.597077	1.863421
44	1	0	-0.016215	5.407070	0.410556
45	1	0	6.061919	-0.923232	-1.299979
46	1	0	5.750380	-0.587837	0.420529
47	1	0	6.655781	-2.058121	-0.048581

Table S4. The coordinate for the lowest-energy conformer of compound **2** for ECD calculation

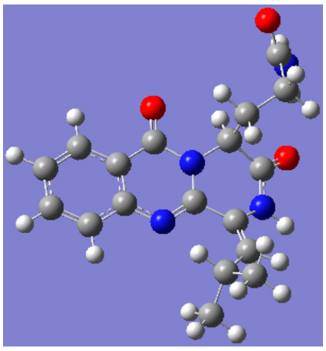

E=-1142.4900148 a.u.

Standard orientation:

Center Number	Atomic Number	Atomic Type	Coordinates (Angstroms) X	Y	Z
1	6	0	4.101073	-3.000198	0.171249
2	6	0	3.113465	-3.999989	0.137728
3	6	0	1.781447	-3.651130	0.001691
4	6	0	1.420462	-2.299242	-0.103004
5	6	0	2.406535	-1.291832	-0.071903
6	6	0	3.757627	-1.664517	0.067172
7	6	0	0.014292	-1.921530	-0.232966
8	7	0	-0.201420	-0.522696	-0.319282
9	6	0	0.848822	0.383222	-0.309801
10	7	0	2.094322	0.047957	-0.196846
11	6	0	-1.610643	-0.078121	-0.345740
12	6	0	-1.781794	1.156143	-1.215851
13	7	0	-0.721111	2.003217	-1.227797
14	6	0	0.488675	1.808635	-0.506974
15	8	0	-0.925740	-2.696024	-0.256802
16	6	0	1.210128	2.876729	-0.124906
17	8	0	-2.821239	1.391913	-1.818538
18	6	0	-2.135347	0.155366	1.094560
19	6	0	-3.597052	0.628500	1.196301
20	6	0	-4.610105	-0.421129	0.732658
21	8	0	-5.057393	-1.259271	1.499021
22	7	0	-4.966861	-0.344592	-0.579084
23	6	0	2.528293	2.949947	0.593127
24	6	0	3.662122	3.253946	-0.405647
25	6	0	2.463865	4.019048	1.696726
26	1	0	5.144368	-3.279542	0.277840
27	1	0	3.396873	-5.043740	0.219044

28	1	0	0.999088	-4.400622	-0.024472
29	1	0	4.509169	-0.883338	0.085208
30	1	0	-2.174321	-0.886788	-0.806001
31	1	0	-0.851486	2.872025	-1.729945
32	1	0	0.781728	3.847820	-0.380945
33	1	0	-1.491475	0.890619	1.588911
34	1	0	-2.020741	-0.787744	1.634579
35	1	0	-3.736671	1.567367	0.654263
36	1	0	-3.815296	0.819439	2.248377
37	1	0	-5.574283	-1.060959	-0.946441
38	1	0	-4.494585	0.278946	-1.222141
39	1	0	2.739747	1.981335	1.046620
40	1	0	4.620592	3.329074	0.116979
41	1	0	3.743687	2.464176	-1.154574
42	1	0	3.493802	4.203550	-0.924723
43	1	0	3.414148	4.072519	2.235136
44	1	0	1.679341	3.799352	2.426020
45	1	0	2.265589	5.013287	1.281344

Table S5. The coordinate for the lowest-energy conformer of compound **3** for ECD calculation.

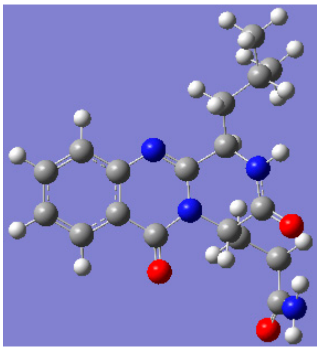

E=-1144.293861 a.u.

Standard orientation:

Center Number	Atomic Number	Atomic Type	Coordinates (Angstroms) X	Y	Z
1	6	0	4.423436	-2.950361	0.212409
2	6	0	3.506887	-3.992575	-0.008359
3	6	0	2.162691	-3.707731	-0.170130
4	6	0	1.718811	-2.377411	-0.113785
5	6	0	2.634125	-1.328691	0.105156
6	6	0	3.997563	-1.635072	0.268209
7	6	0	0.297529	-2.070236	-0.269743
8	7	0	-0.006347	-0.687688	-0.173477
9	6	0	0.979554	0.261294	0.020095
10	7	0	2.233416	-0.003430	0.155389
11	6	0	-1.438129	-0.322385	-0.262526
12	6	0	-1.628242	1.014425	-0.968238
13	7	0	-0.626988	1.906060	-0.797913
14	6	0	0.509050	1.709409	0.105639
15	8	0	-0.586106	-2.887654	-0.456798
16	1	0	0.176698	1.869185	1.141198
17	6	0	1.626342	2.711025	-0.207205
18	6	0	1.273730	4.197223	0.003518
19	6	0	0.888948	4.517866	1.454095
20	6	0	2.455745	5.066431	-0.447980
21	8	0	-2.634283	1.253942	-1.627363
22	1	0	-1.901431	-1.083299	-0.887216
23	6	0	-2.102556	-0.355296	1.136991
24	6	0	-3.601318	-0.001139	1.163138
25	6	0	-4.484072	-1.028814	0.449874
26	8	0	-4.934468	-1.999807	1.036897
27	7	0	-4.724495	-0.773756	-0.865354

28	1	0	5.476597	-3.179548	0.339816
29	1	0	3.854370	-5.019169	-0.050698
30	1	0	1.433434	-4.491468	-0.338915
31	1	0	4.693247	-0.820541	0.435434
32	1	0	-0.758277	2.812143	-1.226253
33	1	0	2.481634	2.450221	0.418901
34	1	0	1.949975	2.549602	-1.241024
35	1	0	0.420147	4.466061	-0.636323
36	1	0	0.697453	5.587282	1.577566
37	1	0	-0.014821	3.993375	1.776947
38	1	0	1.695543	4.246917	2.143729
39	1	0	2.223279	6.130402	-0.350513
40	1	0	2.719109	4.877474	-1.492628
41	1	0	3.344800	4.865368	0.158897
42	1	0	-1.961341	-1.362484	1.536680
43	1	0	-1.570271	0.330011	1.805568
44	1	0	-3.925723	0.006993	2.205116
45	1	0	-3.768455	0.999815	0.756748
46	1	0	-4.239313	-0.033527	-1.358526
47	1	0	-5.237462	-1.462734	-1.393865

Table S6. The coordinate for the lowest-energy conformer of compound **4** for ECD calculation.

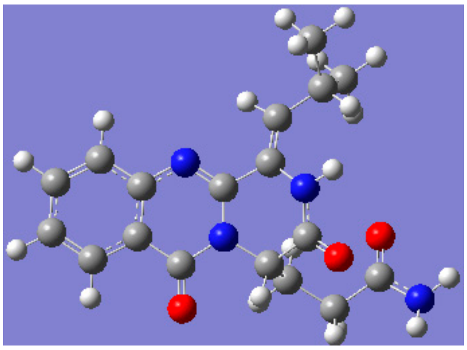

E=-1142.4931498 a.u.

Standard orientation:

Center Number	Atomic Number	Atomic Type	Coordinates (Angstroms) X	Y	Z
1	6	0	-5.407963	0.744444	-0.541343
2	6	0	-5.516841	-0.645553	-0.356043
3	6	0	-4.389227	-1.393137	-0.068823
4	6	0	-3.139834	-0.762258	0.040315
5	6	0	-3.024382	0.632639	-0.140044
6	6	0	-4.183858	1.377942	-0.435029
7	6	0	-1.938728	-1.547877	0.310584
8	7	0	-0.753275	-0.779090	0.395288
9	6	0	-0.759827	0.598043	0.243878
10	7	0	-1.821479	1.293179	-0.014296
11	6	0	0.509044	-1.537621	0.520838
12	6	0	1.562531	-0.765715	1.296450
13	7	0	1.495032	0.588828	1.185432
14	6	0	0.537649	1.295594	0.427960
15	8	0	-1.900772	-2.761691	0.439151
16	6	0	0.739371	2.532612	-0.051440
17	6	0	1.986193	3.365632	0.042918
18	6	0	2.567608	3.601054	-1.364873
19	6	0	1.689787	4.700360	0.751202
20	8	0	2.431747	-1.337648	1.934071
21	1	0	0.275465	-2.428612	1.100751
22	6	0	1.011343	-1.967290	-0.879954
23	6	0	2.317315	-2.766803	-0.871297
24	6	0	3.567966	-1.887405	-0.827541
25	8	0	3.585532	-0.756865	-1.287722
26	7	0	4.687673	-2.491658	-0.330150
27	1	0	-6.295637	1.326618	-0.767784

28	1	0	-6.484289	-1.128892	-0.439590
29	1	0	-4.442391	-2.466266	0.073671
30	1	0	-4.085665	2.449338	-0.568782
31	1	0	2.269864	1.096251	1.589410
32	1	0	-0.105165	2.980526	-0.565418
33	1	0	2.753691	2.840511	0.623971
34	1	0	3.468526	4.219397	-1.310576
35	1	0	2.831442	2.657046	-1.846400
36	1	0	1.845769	4.119030	-2.004372
37	1	0	2.596064	5.308710	0.824763
38	1	0	1.303645	4.543647	1.761795
39	1	0	0.944599	5.279932	0.197458
40	1	0	0.216962	-2.574573	-1.318498
41	1	0	1.142327	-1.084007	-1.509811
42	1	0	2.328118	-3.501766	-0.061420
43	1	0	2.383902	-3.338055	-1.804349
44	1	0	4.599147	-3.273201	0.299749
45	1	0	5.497026	-1.902767	-0.199034

Table S7. The coordinate for the lowest-energy conformer of compound **3** for ECD calculation.

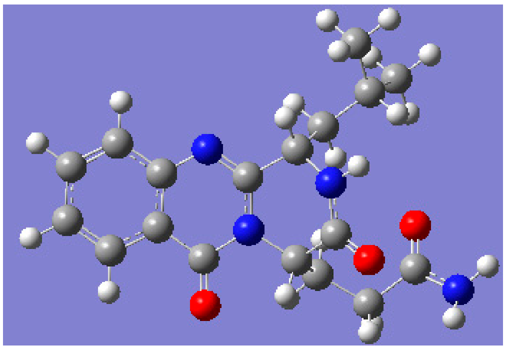

E=-1144.2721485 a.u.

Standard orientation:

Center Number	Atomic Number	Atomic Type	Coordinates (Angstroms) X	Y	Z
1	6	0	-5.474130	0.926211	-0.468557
2	6	0	-5.571434	-0.474214	-0.551403
3	6	0	-4.448316	-1.256609	-0.354057
4	6	0	-3.213557	-0.649939	-0.071979
5	6	0	-3.111582	0.753625	0.016309
6	6	0	-4.264819	1.535319	-0.187674
7	6	0	-2.018612	-1.467768	0.121540
8	7	0	-0.840531	-0.721862	0.375487
9	6	0	-0.864384	0.653706	0.476390
10	7	0	-1.917987	1.383645	0.315177
11	6	0	0.409044	-1.505134	0.512071
12	6	0	1.432789	-0.831052	1.419442
13	7	0	1.308047	0.505519	1.588250
14	6	0	0.427803	1.374769	0.809983
15	8	0	-1.979663	-2.687014	0.069895
16	1	0	0.121818	2.192537	1.463431
17	6	0	1.102693	1.967584	-0.449444
18	6	0	2.319179	2.870272	-0.169929
19	6	0	3.010471	3.221456	-1.494157
20	6	0	1.950122	4.141726	0.606534
21	8	0	2.298926	-1.499542	1.967716
22	1	0	0.128915	-2.427025	1.021292
23	6	0	0.959203	-1.883487	-0.885549
24	6	0	2.287064	-2.646021	-0.872492
25	6	0	3.507080	-1.729516	-0.790642
26	8	0	3.509485	-0.608703	-1.278314
27	7	0	4.621233	-2.284393	-0.229587

28	1	0	-6.358583	1.535633	-0.624925
29	1	0	-6.527047	-0.938504	-0.770087
30	1	0	-4.493404	-2.337881	-0.413077
31	1	0	-4.175120	2.613399	-0.116106
32	1	0	2.021239	0.934333	2.162235
33	1	0	1.421959	1.149010	-1.099524
34	1	0	0.340730	2.539753	-0.990182
35	1	0	3.041938	2.295282	0.423732
36	1	0	3.892686	3.845632	-1.323043
37	1	0	3.334507	2.319993	-2.019543
38	1	0	2.337400	3.778356	-2.155678
39	1	0	2.831990	4.768796	0.765618
40	1	0	1.525752	3.932718	1.593333
41	1	0	1.216341	4.740070	0.055794
42	1	0	0.191809	-2.499319	-1.357084
43	1	0	1.078159	-0.985442	-1.494216
44	1	0	2.308301	-3.399221	-0.081272
45	1	0	2.385267	-3.188589	-1.819894
46	1	0	4.516221	-3.030992	0.439438
47	1	0	5.402139	-1.662385	-0.078228

Table S8. The coordinate for the conformer C1 of compound **3** for NMR calculation.

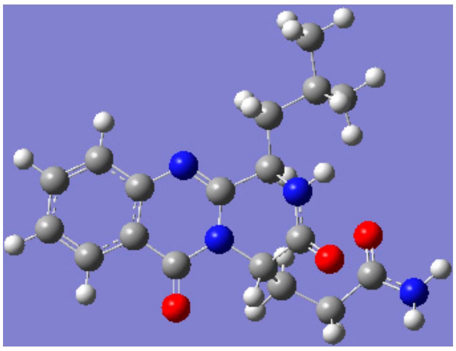

E=-1144.2460465 a.u.

Standard orientation:

Center Number	Atomic Number	Atomic Type	Coordinates (Angstroms) X	Y	Z
1	6	0	-5.399386	0.822946	-0.495915
2	6	0	-5.548607	-0.554476	-0.258338
3	6	0	-4.436085	-1.334417	0.001479
4	6	0	-3.161092	-0.747469	0.030152
5	6	0	-3.006304	0.633991	-0.204133
6	6	0	-4.148255	1.412569	-0.470091
7	6	0	-1.977856	-1.567756	0.286793
8	7	0	-0.766518	-0.839652	0.293722
9	6	0	-0.729830	0.524832	0.066132
10	7	0	-1.767354	1.251618	-0.173110
11	6	0	0.475748	-1.617535	0.497881
12	6	0	1.515737	-0.826125	1.287249
13	7	0	1.436590	0.519076	1.154787
14	6	0	0.655998	1.156157	0.089914
15	8	0	-1.981649	-2.774506	0.473379
16	1	0	1.127225	0.932757	-0.877106
17	6	0	0.615053	2.675038	0.277722
18	6	0	1.978923	3.390331	0.196567
19	6	0	2.698276	3.154377	-1.138421
20	6	0	1.778848	4.890721	0.451962
21	8	0	2.340972	-1.399258	1.983340
22	1	0	0.201382	-2.465809	1.122570
23	6	0	0.998637	-2.155631	-0.857535
24	6	0	2.364197	-2.842856	-0.792405
25	6	0	3.530594	-1.854412	-0.799917
26	8	0	3.431499	-0.732648	-1.276107
27	7	0	4.711898	-2.341739	-0.322954
28	1	0	-6.275154	1.430430	-0.701083

29	1	0	-6.535627	-1.003866	-0.280076
30	1	0	-4.521087	-2.399234	0.184911
31	1	0	-4.017217	2.473830	-0.649105
32	1	0	2.191565	1.045380	1.572702
33	1	0	-0.049295	3.081339	-0.488085
34	1	0	0.136809	2.893656	1.238450
35	1	0	2.631920	3.017994	0.999866
36	1	0	3.622307	3.737895	-1.185786
37	1	0	2.970941	2.107592	-1.293104
38	1	0	2.070998	3.467687	-1.980521
39	1	0	2.735077	5.421507	0.450275
40	1	0	1.296178	5.076833	1.416040
41	1	0	1.150891	5.340202	-0.324727
42	1	0	0.249448	-2.863025	-1.219162
43	1	0	1.055195	-1.341409	-1.582656
44	1	0	2.433003	-3.515530	0.066918
45	1	0	2.485783	-3.467493	-1.684928
46	1	0	4.714049	-3.124506	0.311600
47	1	0	5.469137	-1.681755	-0.223559

Table S9. The coordinate for the conformer C2 of compound **3** for NMR calculation.

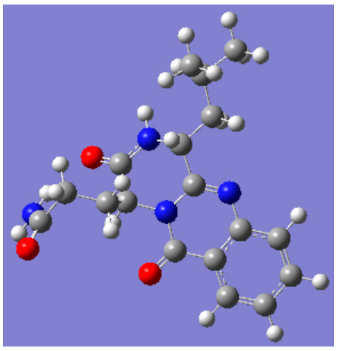

E=-1144.2455425 a.u.

Standard orientation:

Center Number	Atomic Number	Atomic Type	Coordinates (Angstroms) X	Y	Z
1	6	0	4.423436	-2.950361	0.212409
2	6	0	3.506887	-3.992575	-0.008359
3	6	0	2.162691	-3.707731	-0.170130
4	6	0	1.718811	-2.377411	-0.113785
5	6	0	2.634125	-1.328691	0.105156
6	6	0	3.997563	-1.635072	0.268209
7	6	0	0.297529	-2.070236	-0.269743
8	7	0	-0.006347	-0.687688	-0.173477
9	6	0	0.979554	0.261294	0.020095
10	7	0	2.233416	-0.003430	0.155389
11	6	0	-1.438129	-0.322385	-0.262526
12	6	0	-1.628242	1.014425	-0.968238
13	7	0	-0.626988	1.906060	-0.797913
14	6	0	0.509050	1.709409	0.105639
15	8	0	-0.586106	-2.887654	-0.456798
16	1	0	0.176698	1.869185	1.141198
17	6	0	1.626342	2.711025	-0.207205
18	6	0	1.273730	4.197223	0.003518
19	6	0	0.888948	4.517866	1.454095
20	6	0	2.455745	5.066431	-0.447980
21	8	0	-2.634283	1.253942	-1.627363
22	1	0	-1.901431	-1.083299	-0.887216
23	6	0	-2.102556	-0.355296	1.136991
24	6	0	-3.601318	-0.001139	1.163138
25	6	0	-4.484072	-1.028814	0.449874
26	8	0	-4.934468	-1.999807	1.036897
27	7	0	-4.724495	-0.773756	-0.865354
28	1	0	5.476597	-3.179548	0.339816

29	1	0	3.854370	-5.019169	-0.050698
30	1	0	1.433434	-4.491468	-0.338915
31	1	0	4.693247	-0.820541	0.435434
32	1	0	-0.758277	2.812143	-1.226253
33	1	0	2.481634	2.450221	0.418901
34	1	0	1.949975	2.549602	-1.241024
35	1	0	0.420147	4.466061	-0.636323
36	1	0	0.697453	5.587282	1.577566
37	1	0	-0.014821	3.993375	1.776947
38	1	0	1.695543	4.246917	2.143729
39	1	0	2.223279	6.130402	-0.350513
40	1	0	2.719109	4.877474	-1.492628
41	1	0	3.344800	4.865368	0.158897
42	1	0	-1.961341	-1.362484	1.536680
43	1	0	-1.570271	0.330011	1.805568
44	1	0	-3.925723	0.006993	2.205116
45	1	0	-3.768455	0.999815	0.756748
46	1	0	-4.239313	-0.033527	-1.358526
47	1	0	-5.237462	-1.462734	-1.393865

Table S10. The coordinate for the conformer C3 of compound **3** for NMR calculation.

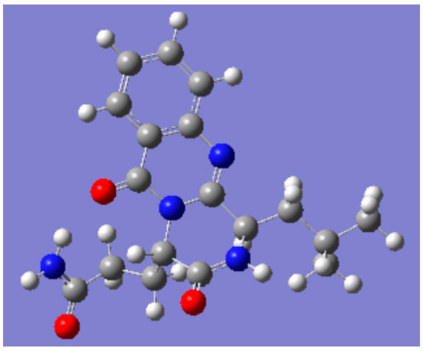

E=-1144.2447972 a.u.

Standard orientation:

Center Number	Atomic Number	Atomic Type	Coordinates (Angstroms) X	Y	Z
1	6	0	-0.527385	5.022303	-0.359329
2	6	0	-1.841234	4.698370	0.022851
3	6	0	-2.175901	3.386426	0.303653
4	6	0	-1.200214	2.380608	0.206185
5	6	0	0.118881	2.701823	-0.170515
6	6	0	0.442970	4.041366	-0.454338
7	6	0	-1.546545	0.988846	0.476361
8	7	0	-0.483112	0.079272	0.317494
9	6	0	0.795499	0.515015	-0.001574
10	7	0	1.109468	1.739836	-0.247313
11	6	0	-0.758928	-1.364411	0.552453
12	6	0	0.393430	-2.024642	1.312868
13	7	0	1.623622	-1.536023	1.003860
14	6	0	1.872207	-0.563861	-0.060159
15	8	0	-2.665250	0.606318	0.802802
16	1	0	1.775812	-1.055880	-1.038403
17	6	0	3.288115	0.012512	0.050083
18	6	0	4.436647	-1.002110	-0.122433
19	6	0	4.419989	-1.692615	-1.492819
20	6	0	5.777537	-0.291860	0.109882
21	8	0	0.199390	-2.924587	2.108018
22	1	0	-1.622949	-1.406917	1.211003
23	6	0	-1.046556	-2.145937	-0.751097
24	6	0	-2.388271	-1.825148	-1.438494
25	6	0	-3.567161	-2.531609	-0.765088
26	8	0	-3.827288	-3.698513	-1.016423
27	7	0	-4.282785	-1.781746	0.114124
28	1	0	-0.271624	6.053805	-0.579174

29	1	0	-2.591056	5.478547	0.096271
30	1	0	-3.181225	3.109541	0.598158
31	1	0	1.461810	4.276134	-0.740856
32	1	0	2.409745	-1.992681	1.444411
33	1	0	3.384102	0.795241	-0.705039
34	1	0	3.379930	0.515310	1.018471
35	1	0	4.353237	-1.783129	0.647079
36	1	0	5.281792	-2.356293	-1.603523
37	1	0	3.526093	-2.303566	-1.646403
38	1	0	4.465410	-0.957500	-2.303671
39	1	0	6.613347	-0.993107	0.037123
40	1	0	5.820568	0.175406	1.097939
41	1	0	5.939667	0.494089	-0.635251
42	1	0	-0.233583	-1.968760	-1.461865
43	1	0	-1.020954	-3.212221	-0.509576
44	1	0	-2.549323	-0.746752	-1.505789
45	1	0	-2.350160	-2.215260	-2.457400
46	1	0	-3.962101	-0.870464	0.415313
47	1	0	-5.012181	-2.241113	0.637915

Table S11. The coordinate for the conformer C4 of compound **3** for NMR calculation.

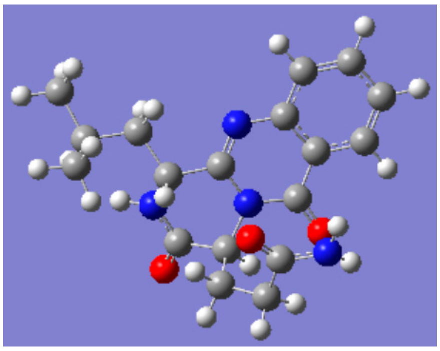

E=-1144.2444319 a.u.

Standard orientation:

Center Number	Atomic Number	Atomic Type	Coordinates (Angstroms) X	Y	Z
1	6	0	3.340116	3.445290	0.092548
2	6	0	4.210436	2.460025	-0.406419
3	6	0	3.720976	1.208184	-0.732988
4	6	0	2.355374	0.925208	-0.567307
5	6	0	1.478124	1.912256	-0.075900
6	6	0	1.992592	3.179889	0.255475
7	6	0	1.833273	-0.402013	-0.886859
8	7	0	0.443482	-0.540884	-0.685413
9	6	0	-0.331600	0.511973	-0.227242
10	7	0	0.123648	1.678055	0.075615
11	6	0	-0.169687	-1.844302	-1.046388
12	6	0	-1.531889	-1.645986	-1.712537
13	7	0	-2.244231	-0.599407	-1.222253
14	6	0	-1.817217	0.213211	-0.078992
15	8	0	2.510084	-1.342736	-1.281110
16	1	0	-1.923979	-0.366724	0.847622
17	6	0	-2.667867	1.481193	0.036176
18	6	0	-4.167091	1.254376	0.316116
19	6	0	-4.420738	0.510087	1.633802
20	6	0	-4.894166	2.606434	0.308441
21	8	0	-1.932874	-2.398398	-2.583291
22	1	0	0.490161	-2.282989	-1.791444
23	6	0	-0.331179	-2.837440	0.128355
24	6	0	0.957983	-3.177844	0.881858
25	6	0	1.338302	-2.121250	1.917546
26	8	0	0.500106	-1.545322	2.594739
27	7	0	2.676998	-1.919789	2.082831
28	1	0	3.729570	4.425339	0.349109

29	1	0	5.263948	2.683797	-0.535850
30	1	0	4.368950	0.431365	-1.122196
31	1	0	1.307752	3.930218	0.634080
32	1	0	-3.174248	-0.473503	-1.596095
33	1	0	-2.240725	2.089158	0.836345
34	1	0	-2.546114	2.064746	-0.882470
35	1	0	-4.603435	0.654812	-0.496282
36	1	0	-5.493344	0.415091	1.824807
37	1	0	-4.004264	-0.500728	1.633337
38	1	0	-3.981557	1.048369	2.480371
39	1	0	-5.969347	2.478430	0.462095
40	1	0	-4.754402	3.134277	-0.639565
41	1	0	-4.521477	3.256776	1.106889
42	1	0	-1.065529	-2.469771	0.849084
43	1	0	-0.746656	-3.747833	-0.311654
44	1	0	0.791964	-4.099936	1.450728
45	1	0	1.778459	-3.375709	0.189387
46	1	0	3.334704	-2.209569	1.376700
47	1	0	2.967299	-1.195531	2.722722

Table S12. The coordinate for the conformer C5 of compound **3** for NMR calculation.

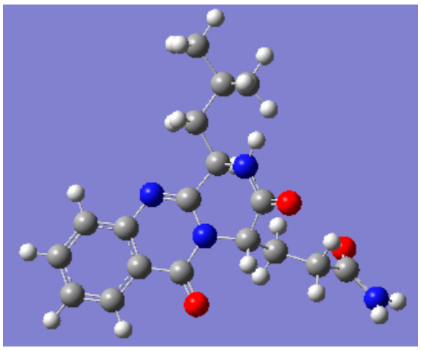

E=-1144.243994 a.u.

Standard orientation:

Center Number	Atomic Number	Atomic Type	Coordinates (Angstroms) X	Y	Z
1	6	0	-5.143445	-1.389299	-0.808823
2	6	0	-4.711388	-2.655489	-0.377446
3	6	0	-3.411470	-2.828438	0.062678
4	6	0	-2.528103	-1.737474	0.080219
5	6	0	-2.957252	-0.465325	-0.348452
6	6	0	-4.281997	-0.307012	-0.796256
7	6	0	-1.148902	-1.913492	0.532279
8	7	0	-0.378880	-0.725994	0.507415
9	6	0	-0.906655	0.475977	0.075184
10	7	0	-2.117742	0.635602	-0.336639
11	6	0	1.033718	-0.853269	0.933919
12	6	0	1.523848	0.405539	1.642280
13	7	0	0.950879	1.560373	1.218489
14	6	0	0.052624	1.660988	0.067212
15	8	0	-0.655544	-2.967326	0.900848
16	1	0	0.637477	1.579275	-0.860463
17	6	0	-0.676521	3.008749	0.065405
18	6	0	0.219045	4.254339	-0.090568
19	6	0	1.013262	4.256277	-1.403578
20	6	0	-0.647322	5.516806	0.020770
21	8	0	2.382080	0.355057	2.508910
22	1	0	1.052434	-1.658146	1.666118
23	6	0	1.939546	-1.233115	-0.259451
24	6	0	3.341148	-1.670006	0.170195
25	6	0	4.273928	-1.827277	-1.026756
26	8	0	4.178885	-1.142838	-2.032001
27	7	0	5.245990	-2.774982	-0.884394
28	1	0	-6.163963	-1.259423	-1.154787

29	1	0	-5.397748	-3.495325	-0.391249
30	1	0	-3.050410	-3.793767	0.397937
31	1	0	-4.601105	0.675791	-1.124416
32	1	0	1.308884	2.410042	1.632014
33	1	0	-1.404794	2.984856	-0.747576
34	1	0	-1.258668	3.084302	0.990061
35	1	0	0.941397	4.291450	0.738054
36	1	0	1.591128	5.178981	-1.505984
37	1	0	1.722836	3.427062	-1.469365
38	1	0	0.343832	4.190480	-2.268040
39	1	0	-0.036805	6.421787	-0.043391
40	1	0	-1.192719	5.549598	0.968466
41	1	0	-1.385313	5.558082	-0.787285
42	1	0	1.450805	-2.050584	-0.795465
43	1	0	2.023238	-0.400994	-0.962434
44	1	0	3.789217	-0.923255	0.834517
45	1	0	3.287570	-2.597883	0.748687
46	1	0	5.352788	-3.319402	-0.045058
47	1	0	5.940426	-2.867933	-1.609341

Table S13. The coordinate for the conformer C6 of compound **3** for NMR calculation.

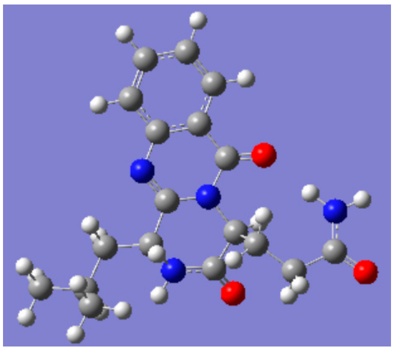

E=-1144.2433829 a.u.

Standard orientation:

Center Number	Atomic Number	Atomic Type	Coordinates (Angstroms) X	Y	Z
1	6	0	-1.721192	4.917395	0.225409
2	6	0	-0.353539	5.168125	0.014945
3	6	0	0.526090	4.113261	-0.143631
4	6	0	0.047535	2.793418	-0.098514
5	6	0	-1.322619	2.536771	0.111325
6	6	0	-2.203429	3.622100	0.274669
7	6	0	0.961998	1.669370	-0.254298
8	7	0	0.346665	0.403460	-0.224727
9	6	0	-1.012513	0.263684	-0.001382
10	7	0	-1.826264	1.249412	0.162485
11	6	0	1.249204	-0.761451	-0.396316
12	6	0	0.541954	-1.950626	-1.039945
13	7	0	-0.796764	-2.022024	-0.821143
14	6	0	-1.534924	-1.163654	0.103305
15	8	0	2.176879	1.767339	-0.395938
16	1	0	-1.341404	-1.485447	1.137804
17	6	0	-3.043088	-1.254820	-0.159388
18	6	0	-3.676932	-2.645045	0.045839
19	6	0	-3.506311	-3.174242	1.476075
20	6	0	-5.162885	-2.584256	-0.334803
21	8	0	1.162301	-2.771173	-1.692714
22	1	0	2.010966	-0.449815	-1.107707
23	6	0	1.919631	-1.150899	0.947501
24	6	0	3.168417	-2.033085	0.811887
25	6	0	4.504390	-1.395288	0.414925
26	8	0	5.518276	-2.076598	0.426463
27	7	0	4.520493	-0.076982	0.079812
28	1	0	-2.405701	5.750126	0.350887

29	1	0	0.009555	6.189447	-0.021643
30	1	0	1.584884	4.278196	-0.304775
31	1	0	-3.254876	3.413678	0.436442
32	1	0	-1.258181	-2.839796	-1.195035
33	1	0	-3.534032	-0.534684	0.498014
34	1	0	-3.232865	-0.910290	-1.181282
35	1	0	-3.205332	-3.366271	-0.637468
36	1	0	-4.017141	-4.133111	1.598461
37	1	0	-2.458416	-3.335720	1.744415
38	1	0	-3.934216	-2.478872	2.206230
39	1	0	-5.633946	-3.567065	-0.246393
40	1	0	-5.301872	-2.241114	-1.364022
41	1	0	-5.707972	-1.896301	0.320040
42	1	0	2.162417	-0.230831	1.485249
43	1	0	1.185224	-1.674854	1.569016
44	1	0	3.364838	-2.520333	1.770889
45	1	0	2.993639	-2.845269	0.101306
46	1	0	3.705256	0.515019	-0.019847
47	1	0	5.409881	0.305168	-0.203673

Table S14. The coordinate for the conformer C7 of compound **3** for NMR calculation.

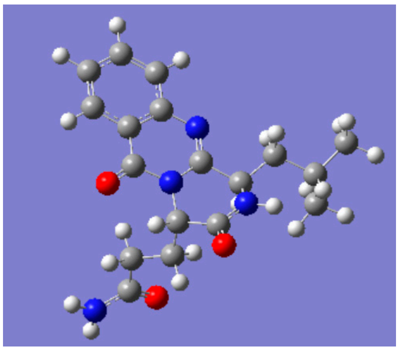

E=-1144.2428681 a.u.

Standard orientation:

Center Number	Atomic Number	Atomic Type	Coordinates (Angstroms) X	Y	Z
1	6	0	-2.266329	4.440534	-0.929245
2	6	0	-3.356688	3.856669	-0.261230
3	6	0	-3.215472	2.619153	0.340606
4	6	0	-1.983032	1.949103	0.282199
5	6	0	-0.885593	2.533128	-0.381360
6	6	0	-1.045843	3.792197	-0.989331
7	6	0	-1.831203	0.632116	0.900009
8	7	0	-0.545111	0.064099	0.735616
9	6	0	0.477960	0.752819	0.106682
10	7	0	0.351432	1.914416	-0.436901
11	6	0	-0.319923	-1.281904	1.316857
12	6	0	1.061737	-1.382794	1.963614
13	7	0	2.026037	-0.655539	1.340907
14	6	0	1.826320	0.042065	0.068959
15	8	0	-2.710447	0.032050	1.497857
16	1	0	1.772281	-0.691906	-0.747451
17	6	0	2.989684	0.996721	-0.217429
18	6	0	4.368524	0.331712	-0.403250
19	6	0	4.394470	-0.672849	-1.563290
20	6	0	5.432051	1.420121	-0.604282
21	8	0	1.260070	-2.082534	2.940561
22	1	0	-1.052650	-1.389640	2.113369
23	6	0	-0.493242	-2.429166	0.298615
24	6	0	-1.898753	-2.536734	-0.296304
25	6	0	-1.969080	-3.628872	-1.359741
26	8	0	-1.046419	-3.863819	-2.122243
27	7	0	-3.150374	-4.309781	-1.416747
28	1	0	-2.382606	5.411270	-1.400563

29	1	0	-4.307242	4.377452	-0.218675
30	1	0	-4.039326	2.145899	0.862049
31	1	0	-0.193931	4.231774	-1.495727
32	1	0	2.964573	-0.757182	1.700917
33	1	0	2.736951	1.558712	-1.118877
34	1	0	3.039074	1.732854	0.591838
35	1	0	4.638748	-0.210180	0.514893
36	1	0	5.400338	-1.078603	-1.702520
37	1	0	3.727313	-1.523554	-1.400140
38	1	0	4.100011	-0.195136	-2.504024
39	1	0	6.431224	0.984556	-0.692212
40	1	0	5.448748	2.126142	0.231081
41	1	0	5.239310	1.992694	-1.517670
42	1	0	0.227909	-2.338808	-0.517005
43	1	0	-0.245948	-3.355268	0.826035
44	1	0	-2.640392	-2.694619	0.490850
45	1	0	-2.177249	-1.600985	-0.793171
46	1	0	-3.922034	-4.108697	-0.803342
47	1	0	-3.278158	-4.998827	-2.141656

Table S15. The coordinate for the conformer C8 of compound **3** for NMR calculation.

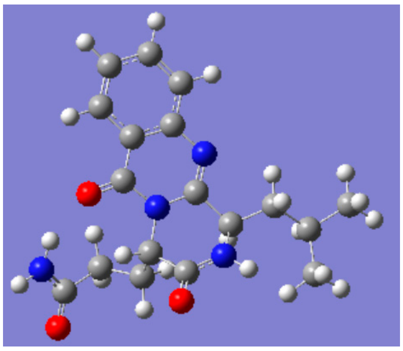

E=-1144.2428074 a.u.

Standard orientation:

Center Number	Atomic Number	Atomic Type	Coordinates (Angstroms) X	Y	Z
1	6	0	-0.724982	5.014538	-0.286268
2	6	0	-2.025115	4.633477	0.089971
3	6	0	-2.307926	3.305326	0.351083
4	6	0	-1.293092	2.340553	0.239988
5	6	0	0.012202	2.719008	-0.130024
6	6	0	0.283439	4.074183	-0.394402
7	6	0	-1.583564	0.932030	0.486914
8	7	0	-0.484500	0.067267	0.315777
9	6	0	0.777117	0.557243	0.008711
10	7	0	1.039301	1.797525	-0.219216
11	6	0	-0.709825	-1.389991	0.520086
12	6	0	0.462757	-2.022189	1.271778
13	7	0	1.674482	-1.482287	0.978335
14	6	0	1.896132	-0.483083	-0.068748
15	8	0	-2.686795	0.500102	0.803618
16	1	0	1.812087	-0.960599	-1.057427
17	6	0	3.302163	0.128387	0.037747
18	6	0	4.418329	-0.638704	-0.698639
19	6	0	5.728186	0.155736	-0.609519
20	6	0	4.628674	-2.076713	-0.204532
21	8	0	0.298905	-2.942430	2.050780
22	1	0	-1.573141	-1.476283	1.175244
23	6	0	-0.967526	-2.154280	-0.799658
24	6	0	-2.319242	-1.868955	-1.483181
25	6	0	-3.472449	-2.632560	-0.827826
26	8	0	-3.686453	-3.803720	-1.101766
27	7	0	-4.220335	-1.927255	0.061435
28	1	0	-0.510021	6.058377	-0.490995

29	1	0	-2.805159	5.382316	0.173965
30	1	0	-3.301776	2.984608	0.639965
31	1	0	1.292102	4.353672	-0.676720
32	1	0	2.474795	-1.910998	1.420694
33	1	0	3.246583	1.138434	-0.367379
34	1	0	3.554240	0.243807	1.099481
35	1	0	4.132280	-0.689513	-1.758763
36	1	0	6.524804	-0.330848	-1.179169
37	1	0	5.611538	1.170397	-1.000779
38	1	0	6.070165	0.238864	0.427842
39	1	0	5.453524	-2.553460	-0.741266
40	1	0	3.748096	-2.707409	-0.354410
41	1	0	4.887444	-2.099018	0.861007
42	1	0	-0.160853	-1.933132	-1.505161
43	1	0	-0.903315	-3.223656	-0.579872
44	1	0	-2.519976	-0.796133	-1.528834
45	1	0	-2.265067	-2.236183	-2.509861
46	1	0	-3.934891	-1.011193	0.382607
47	1	0	-4.933023	-2.424456	0.573581

Table S16. The coordinate for the conformer C9 of compound **3** for NMR calculation.

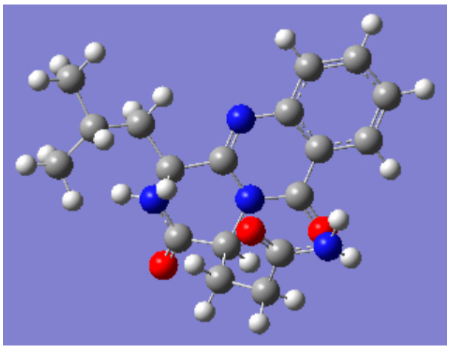

E=-1144.2425858 a.u.

Standard orientation:

Center Number	Atomic Number	Atomic Type	Coordinates (Angstroms) X	Y	Z
1	6	0	3.450967	3.362192	-0.050231
2	6	0	4.290131	2.333382	-0.513292
3	6	0	3.763188	1.084452	-0.788462
4	6	0	2.390871	0.848090	-0.606236
5	6	0	1.544983	1.878199	-0.150622
6	6	0	2.097135	3.142557	0.128202
7	6	0	1.828797	-0.474890	-0.869371
8	7	0	0.436133	-0.565668	-0.658148
9	6	0	-0.307087	0.526081	-0.239411
10	7	0	0.185505	1.687977	0.016834
11	6	0	-0.212942	-1.865201	-0.968651
12	6	0	-1.568660	-1.652333	-1.642114
13	7	0	-2.249596	-0.569596	-1.188809
14	6	0	-1.801837	0.267599	-0.068987
15	8	0	2.476892	-1.449723	-1.226986
16	1	0	-1.908144	-0.288793	0.873879
17	6	0	-2.638530	1.550351	0.035246
18	6	0	-3.939384	1.430309	0.854060
19	6	0	-4.595743	2.812204	0.974050
20	6	0	-4.940480	0.407081	0.300194
21	8	0	-1.989961	-2.421074	-2.489031
22	1	0	0.433379	-2.348215	-1.697961
23	6	0	-0.397744	-2.811805	0.240364
24	6	0	0.883017	-3.156199	1.006461
25	6	0	1.287228	-2.072614	2.004471
26	8	0	0.462538	-1.459647	2.665679
27	7	0	2.629888	-1.888144	2.155744
28	1	0	3.869612	4.339966	0.165773

29	1	0	5.348945	2.521349	-0.655673
30	1	0	4.386501	0.274452	-1.149260
31	1	0	1.436493	3.927035	0.479559
32	1	0	-3.171318	-0.421670	-1.573594
33	1	0	-2.006198	2.310575	0.493548
34	1	0	-2.856624	1.913640	-0.977281
35	1	0	-3.657070	1.106630	1.865227
36	1	0	-5.490506	2.772555	1.601726
37	1	0	-3.912905	3.544540	1.414215
38	1	0	-4.899575	3.191923	-0.007746
39	1	0	-5.851073	0.389141	0.905672
40	1	0	-4.542160	-0.611151	0.297116
41	1	0	-5.243470	0.658531	-0.723705
42	1	0	-1.123386	-2.403430	0.947923
43	1	0	-0.834392	-3.726911	-0.168341
44	1	0	0.694944	-4.053313	1.607443
45	1	0	1.698426	-3.397574	0.321994
46	1	0	3.279172	-2.215299	1.458238
47	1	0	2.936634	-1.149125	2.770565

Table S17. The coordinate for the conformer C1 of compound **5** for NMR calculation.

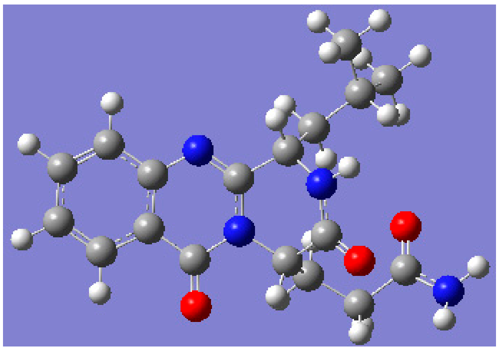

E=-1144.2458631 a.u.

Standard orientation:

Center Number	Atomic Number	Atomic Type	Coordinates (Angstroms) X	Y	Z
1	6	0	-5.474130	0.926211	-0.468557
2	6	0	-5.571434	-0.474214	-0.551403
3	6	0	-4.448316	-1.256609	-0.354057
4	6	0	-3.213557	-0.649939	-0.071979
5	6	0	-3.111582	0.753625	0.016309
6	6	0	-4.264819	1.535319	-0.187674
7	6	0	-2.018612	-1.467768	0.121540
8	7	0	-0.840531	-0.721862	0.375487
9	6	0	-0.864384	0.653706	0.476390
10	7	0	-1.917987	1.383645	0.315177
11	6	0	0.409044	-1.505134	0.512071
12	6	0	1.432789	-0.831052	1.419442
13	7	0	1.308047	0.505519	1.588250
14	6	0	0.427803	1.374769	0.809983
15	8	0	-1.979663	-2.687014	0.069895
16	1	0	0.121818	2.192537	1.463431
17	6	0	1.102693	1.967584	-0.449444
18	6	0	2.319179	2.870272	-0.169929
19	6	0	3.010471	3.221456	-1.494157
20	6	0	1.950122	4.141726	0.606534
21	8	0	2.298926	-1.499542	1.967716
22	1	0	0.128915	-2.427025	1.021292
23	6	0	0.959203	-1.883487	-0.885549
24	6	0	2.287064	-2.646021	-0.872492
25	6	0	3.507080	-1.729516	-0.790642
26	8	0	3.509485	-0.608703	-1.278314
27	7	0	4.621233	-2.284393	-0.229587
28	1	0	-6.358583	1.535633	-0.624925

29	1	0	-6.527047	-0.938504	-0.770087
30	1	0	-4.493404	-2.337881	-0.413077
31	1	0	-4.175120	2.613399	-0.116106
32	1	0	2.021239	0.934333	2.162235
33	1	0	1.421959	1.149010	-1.099524
34	1	0	0.340730	2.539753	-0.990182
35	1	0	3.041938	2.295282	0.423732
36	1	0	3.892686	3.845632	-1.323043
37	1	0	3.334507	2.319993	-2.019543
38	1	0	2.337400	3.778356	-2.155678
39	1	0	2.831990	4.768796	0.765618
40	1	0	1.525752	3.932718	1.593333
41	1	0	1.216341	4.740070	0.055794
42	1	0	0.191809	-2.499319	-1.357084
43	1	0	1.078159	-0.985442	-1.494216
44	1	0	2.308301	-3.399221	-0.081272
45	1	0	2.385267	-3.188589	-1.819894
46	1	0	4.516221	-3.030992	0.439438
47	1	0	5.402139	-1.662385	-0.078228

Table S18. The coordinate for the conformer C2 of compound **5** for NMR calculation.

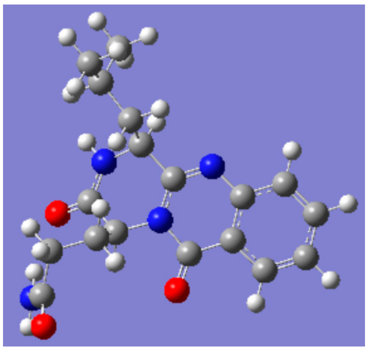

E=-1144.2455392 a.u.

Standard orientation:

Center Number	Atomic Number	Atomic Type	Coordinates (Angstroms) X	Y	Z
1	6	0	5.400768	-0.329043	0.256239
2	6	0	5.083554	-1.696208	0.337851
3	6	0	3.770185	-2.109149	0.205115
4	6	0	2.757616	-1.160511	-0.009489
5	6	0	3.071035	0.211362	-0.094470
6	6	0	4.412395	0.614732	0.041808
7	6	0	1.366650	-1.588945	-0.141023
8	7	0	0.445746	-0.521773	-0.322995
9	6	0	0.871971	0.785320	-0.424397
10	7	0	2.100409	1.170100	-0.325148
11	6	0	-0.983181	-0.905191	-0.397966
12	6	0	-1.792843	0.054944	-1.259050
13	7	0	-1.335457	1.322950	-1.352760
14	6	0	-0.160880	1.868451	-0.676124
15	8	0	0.972492	-2.740744	-0.098430
16	1	0	0.322408	2.554620	-1.372784
17	6	0	-0.497842	2.644964	0.617910
18	6	0	-1.448685	3.842869	0.437352
19	6	0	-1.796061	4.436579	1.809483
20	6	0	-0.871638	4.923512	-0.487433
21	8	0	-2.817781	-0.317686	-1.822214
22	1	0	-1.006625	-1.870122	-0.902123
23	6	0	-1.574576	-1.086284	1.024976
24	6	0	-3.056208	-1.500966	1.091166
25	6	0	-3.323015	-2.914415	0.566498
26	8	0	-3.271053	-3.889176	1.299672
27	7	0	-3.620341	-2.992149	-0.759164
28	1	0	6.433008	-0.010518	0.361086

29	1	0	5.869064	-2.425176	0.505077
30	1	0	3.496130	-3.156021	0.264676
31	1	0	4.642767	1.671857	-0.027694
32	1	0	-1.893449	1.940629	-1.926703
33	1	0	-0.931961	1.950751	1.344989
34	1	0	0.450829	2.990855	1.041705
35	1	0	-2.384988	3.474875	-0.003748
36	1	0	-2.506035	5.262701	1.713559
37	1	0	-2.245643	3.688906	2.469345
38	1	0	-0.902889	4.825646	2.309660
39	1	0	-1.556628	5.772092	-0.566541
40	1	0	-0.692517	4.563984	-1.505298
41	1	0	0.080688	5.303956	-0.103215
42	1	0	-0.967013	-1.842695	1.526665
43	1	0	-1.448223	-0.153474	1.582426
44	1	0	-3.352159	-1.507773	2.141666
45	1	0	-3.684992	-0.772526	0.573594
46	1	0	-3.556340	-2.181115	-1.362748
47	1	0	-3.742678	-3.906631	-1.166019

Table S19. The coordinate for the conformer C3 of compound **5** for NMR calculation.

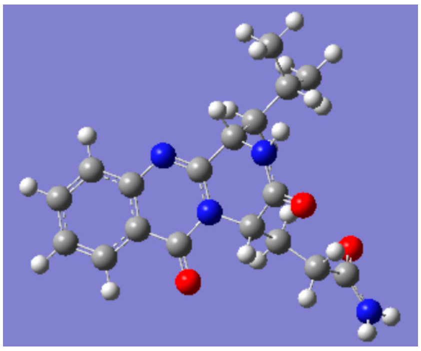

E=-1144.2443127 a.u.

Standard orientation:

Center Number	Atomic Number	Atomic Type	Coordinates (Angstroms) X	Y	Z
1	6	0	-5.426716	-0.080108	-0.875774
2	6	0	-5.226167	-1.462291	-0.712048
3	6	0	-3.995291	-1.936405	-0.296592
4	6	0	-2.949236	-1.035624	-0.038608
5	6	0	-3.146064	0.351369	-0.198923
6	6	0	-4.405046	0.817116	-0.623477
7	6	0	-1.643759	-1.528041	0.393831
8	7	0	-0.681006	-0.509441	0.608629
9	6	0	-0.991794	0.823135	0.439800
10	7	0	-2.144280	1.267910	0.061869
11	6	0	0.663496	-0.973128	1.025567
12	6	0	1.404630	0.058027	1.868784
13	7	0	1.048048	1.354125	1.686258
14	6	0	0.088892	1.853215	0.704754
15	8	0	-1.350473	-2.700452	0.565869
16	1	0	-0.434494	2.694026	1.161516
17	6	0	0.736147	2.336749	-0.615508
18	6	0	1.804413	3.434654	-0.461428
19	6	0	2.444927	3.723289	-1.826134
20	6	0	1.244170	4.723493	0.156336
21	8	0	2.280624	-0.287357	2.647428
22	1	0	0.491958	-1.835056	1.669041
23	6	0	1.499194	-1.424593	-0.195531
24	6	0	2.722567	-2.258659	0.188119
25	6	0	3.651531	-2.471749	-1.002812
26	8	0	3.771752	-1.656118	-1.902225
27	7	0	4.365401	-3.635221	-0.980713
28	1	0	-6.394668	0.286549	-1.202447

29	1	0	-6.037016	-2.154390	-0.912234
30	1	0	-3.812016	-2.996285	-0.163486
31	1	0	-4.545520	1.885612	-0.741867
32	1	0	1.570628	2.023488	2.234620
33	1	0	1.179721	1.479094	-1.130756
34	1	0	-0.074211	2.702171	-1.255491
35	1	0	2.596546	3.051927	0.195584
36	1	0	3.237845	4.471598	-1.738956
37	1	0	2.885621	2.822365	-2.261838
38	1	0	1.704539	4.108337	-2.535695
39	1	0	2.019483	5.491870	0.221560
40	1	0	0.854694	4.577307	1.168558
41	1	0	0.428997	5.129344	-0.452220
42	1	0	0.845627	-2.015194	-0.841223
43	1	0	1.825525	-0.560983	-0.777549
44	1	0	3.301146	-1.750442	0.966517
45	1	0	2.409765	-3.217224	0.614837
46	1	0	4.301638	-4.294860	-0.223685
47	1	0	5.056450	-3.792143	-1.697811

Table S20. The coordinate for the conformer C4 of compound **5** for NMR calculation.

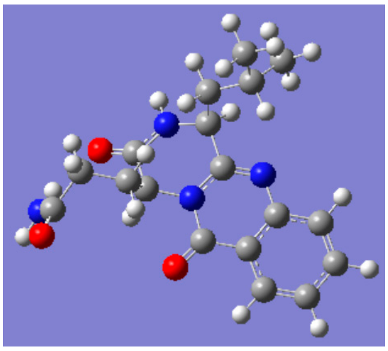

E=-1144.2442476 a.u.

Standard orientation:

Center Number	Atomic Number	Atomic Type	Coordinates (Angstroms) X	Y	Z
1	6	0	4.479926	-2.585860	0.085208
2	6	0	3.583488	-3.636443	0.347174
3	6	0	2.219875	-3.409472	0.297376
4	6	0	1.735746	-2.128414	-0.011806
5	6	0	2.630453	-1.072355	-0.276805
6	6	0	4.014653	-1.320396	-0.224566
7	6	0	0.296395	-1.879850	-0.064051
8	7	0	-0.050402	-0.532875	-0.354933
9	6	0	0.914475	0.416861	-0.622499
10	7	0	2.187314	0.197963	-0.602673
11	6	0	-1.501476	-0.235763	-0.399485
12	6	0	-1.830212	0.886799	-1.376015
13	7	0	-0.845540	1.774962	-1.636319
14	6	0	0.462672	1.819785	-0.983005
15	8	0	-0.572581	-2.713425	0.119560
16	1	0	1.179533	2.164734	-1.728299
17	6	0	0.467250	2.815976	0.205366
18	6	0	1.831503	3.131133	0.845635
19	6	0	2.804559	3.792667	-0.138813
20	6	0	1.621587	4.017508	2.081294
21	8	0	-2.941984	0.966578	-1.889577
22	1	0	-1.973672	-1.134558	-0.794184
23	6	0	-2.056890	0.011687	1.027471
24	6	0	-3.565849	0.303856	1.120650
25	6	0	-4.445765	-0.891942	0.747010
26	8	0	-4.788434	-1.718319	1.577693
27	7	0	-4.810643	-0.953977	-0.562631
28	1	0	5.548788	-2.770053	0.124472

29	1	0	3.962210	-4.623952	0.587542
30	1	0	1.505855	-4.200656	0.494297
31	1	0	4.694327	-0.502040	-0.433770
32	1	0	-1.094920	2.526314	-2.266242
33	1	0	0.025772	3.749426	-0.167684
34	1	0	-0.219565	2.447756	0.973773
35	1	0	2.280255	2.188468	1.176355
36	1	0	3.738036	4.061002	0.363771
37	1	0	3.067067	3.130382	-0.966655
38	1	0	2.383297	4.714911	-0.555985
39	1	0	2.571865	4.224997	2.580964
40	1	0	0.961142	3.542770	2.813320
41	1	0	1.176466	4.981326	1.810144
42	1	0	-1.827087	-0.879987	1.614872
43	1	0	-1.512145	0.843935	1.482246
44	1	0	-3.795096	0.533778	2.162715
45	1	0	-3.828236	1.179080	0.521724
46	1	0	-4.423979	-0.315922	-1.247748
47	1	0	-5.335367	-1.757567	-0.871975

Table S21. The coordinate for the conformer C5 of compound **5** for NMR calculation.

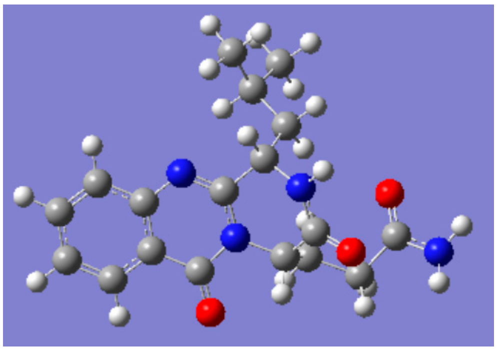

E=-1144.2439025 a.u.

Standard orientation:

Center Number	Atomic Number	Atomic Type	Coordinates (Angstroms) X	Y	Z
1	6	0	-5.335907	-0.172053	-0.234602
2	6	0	-5.126845	-1.511593	-0.607412
3	6	0	-3.853429	-2.049345	-0.563234
4	6	0	-2.772700	-1.254887	-0.147541
5	6	0	-2.977112	0.087951	0.228853
6	6	0	-4.279927	0.619507	0.178975
7	6	0	-1.423886	-1.813925	-0.097739
8	7	0	-0.429500	-0.895427	0.321119
9	6	0	-0.746763	0.397763	0.686568
10	7	0	-1.939925	0.893826	0.661824
11	6	0	0.955206	-1.419665	0.374310
12	6	0	1.808361	-0.727494	1.433025
13	7	0	1.396704	0.496147	1.840118
14	6	0	0.371866	1.299395	1.172308
15	8	0	-1.124649	-2.962814	-0.382757
16	1	0	-0.088155	1.928533	1.934594
17	6	0	0.992071	2.208346	0.080032
18	6	0	0.054963	3.227134	-0.593427
19	6	0	-0.517406	4.247433	0.399176
20	6	0	0.809288	3.936814	-1.726630
21	8	0	2.794898	-1.290543	1.887519
22	1	0	0.866136	-2.454956	0.703818
23	6	0	1.585768	-1.430276	-1.040077
24	6	0	3.030881	-1.934102	-1.096774
25	6	0	4.060326	-0.846188	-0.794154
26	8	0	3.869175	0.323860	-1.088709
27	7	0	5.243949	-1.285832	-0.272297
28	1	0	-6.337453	0.244732	-0.270224

29	1	0	-5.964609	-2.121292	-0.928338
30	1	0	-3.662876	-3.078727	-0.843752
31	1	0	-4.426657	1.652545	0.473453
32	1	0	2.013205	0.961069	2.493740
33	1	0	1.815950	2.750099	0.562633
34	1	0	1.470955	1.579774	-0.675120
35	1	0	-0.786923	2.682887	-1.036114
36	1	0	-1.130608	4.990577	-0.118914
37	1	0	-1.153386	3.777616	1.152764
38	1	0	0.284070	4.789514	0.914976
39	1	0	0.156636	4.639647	-2.252626
40	1	0	1.196670	3.225919	-2.461866
41	1	0	1.662144	4.505115	-1.339561
42	1	0	0.952335	-2.077764	-1.648228
43	1	0	1.547712	-0.430423	-1.475884
44	1	0	3.176319	-2.805050	-0.453133
45	1	0	3.241939	-2.266552	-2.119760
46	1	0	5.255051	-2.138072	0.266101
47	1	0	5.889041	-0.569102	0.027729

Table S22. The coordinate for the conformer C6 of compound **5** for NMR calculation.

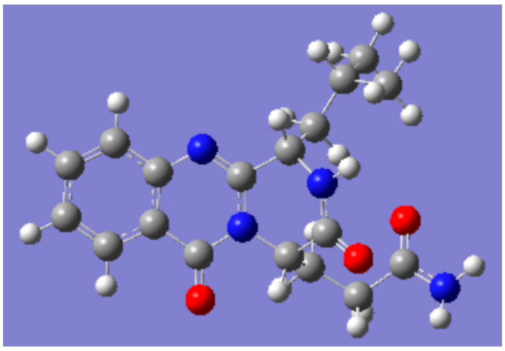

E=-1144.2438034 a.u.

Standard orientation:

Center Number	Atomic Number	Atomic Type	Coordinates (Angstroms) X	Y	Z
1	6	0	-5.505320	0.950561	-0.322466
2	6	0	-5.600535	-0.444346	-0.473306
3	6	0	-4.470169	-1.230626	-0.345221
4	6	0	-3.230440	-0.633604	-0.064422
5	6	0	-3.130266	0.764313	0.091993
6	6	0	-4.290910	1.550089	-0.042921
7	6	0	-2.028943	-1.455261	0.059419
8	7	0	-0.849749	-0.718290	0.331619
9	6	0	-0.872931	0.650697	0.494303
10	7	0	-1.931077	1.384426	0.390042
11	6	0	0.403336	-1.500680	0.424036
12	6	0	1.419043	-0.868003	1.368882
13	7	0	1.301797	0.462937	1.581133
14	6	0	0.430251	1.358292	0.820227
15	8	0	-1.986150	-2.669689	-0.059496
16	1	0	0.137606	2.170714	1.488600
17	6	0	1.092824	1.975075	-0.442386
18	6	0	1.894091	3.268918	-0.196319
19	6	0	3.051803	3.098381	0.797767
20	6	0	2.416624	3.804961	-1.536294
21	8	0	2.275495	-1.562062	1.900405
22	1	0	0.128243	-2.452249	0.878057
23	6	0	0.963855	-1.791385	-0.989899
24	6	0	2.275882	-2.580448	-1.008820
25	6	0	3.513069	-1.707198	-0.805606
26	8	0	3.545726	-0.535700	-1.153184
27	7	0	4.608984	-2.350700	-0.307428
28	1	0	-6.395426	1.563214	-0.424863

29	1	0	-6.560160	-0.901266	-0.689956
30	1	0	-4.513361	-2.307767	-0.457414
31	1	0	-4.202701	2.623624	0.080254
32	1	0	2.030136	0.871311	2.150021
33	1	0	1.742548	1.226998	-0.906139
34	1	0	0.295641	2.206281	-1.155534
35	1	0	1.199636	4.014563	0.216272
36	1	0	3.650860	4.012078	0.852222
37	1	0	2.702488	2.901098	1.816943
38	1	0	3.715849	2.281968	0.497806
39	1	0	2.938186	4.757350	-1.403040
40	1	0	1.602429	3.969468	-2.248348
41	1	0	3.120408	3.101385	-1.992242
42	1	0	0.193125	-2.361330	-1.511710
43	1	0	1.106729	-0.856681	-1.535343
44	1	0	2.255478	-3.402197	-0.288301
45	1	0	2.391387	-3.039647	-1.997543
46	1	0	4.484428	-3.177611	0.255300
47	1	0	5.398621	-1.768718	-0.068219

Table S23. The coordinate for the conformer C7 of compound **5** for NMR calculation.

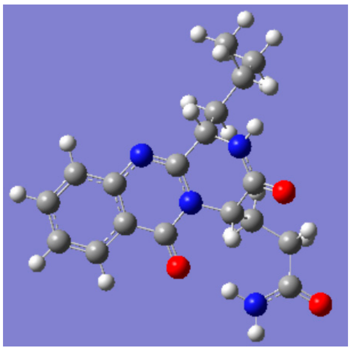

E=-1144.2437512 a.u.

Standard orientation:

Center Number	Atomic Number	Atomic Type	Coordinates (Angstroms) X	Y	Z
1	6	0	-5.304605	-0.387507	-0.291140
2	6	0	-4.934745	-1.742444	-0.369942
3	6	0	-3.609397	-2.106213	-0.222085
4	6	0	-2.636043	-1.119236	0.007846
5	6	0	-3.002039	0.240194	0.088145
6	6	0	-4.356253	0.592913	-0.065267
7	6	0	-1.234731	-1.486126	0.161770
8	7	0	-0.361459	-0.402706	0.391490
9	6	0	-0.831804	0.894422	0.456391
10	7	0	-2.070270	1.233336	0.320289
11	6	0	1.072353	-0.751560	0.563058
12	6	0	1.841740	0.280843	1.382465
13	7	0	1.329117	1.538142	1.413636
14	6	0	0.164787	2.014352	0.675860
15	8	0	-0.798865	-2.631342	0.100591
16	1	0	-0.355314	2.730505	1.313249
17	6	0	0.514181	2.712544	-0.660817
18	6	0	1.454407	3.925896	-0.540815
19	6	0	1.813234	4.441873	-1.941056
20	6	0	0.858780	5.053619	0.313270
21	8	0	2.868673	-0.031802	1.960186
22	1	0	1.089366	-1.665717	1.154470
23	6	0	1.753556	-1.013290	-0.807728
24	6	0	3.081886	-1.779890	-0.740098
25	6	0	3.075147	-3.297295	-0.528973
26	8	0	4.131067	-3.910822	-0.564603
27	7	0	1.882045	-3.917653	-0.326602
28	1	0	-6.346893	-0.108853	-0.408469

29	1	0	-5.690465	-2.499969	-0.546805
30	1	0	-3.296943	-3.142273	-0.278427
31	1	0	-4.626696	1.640686	-0.000336
32	1	0	1.868723	2.205494	1.947863
33	1	0	0.963026	1.977464	-1.337598
34	1	0	-0.430502	3.024735	-1.118821
35	1	0	2.387155	3.590718	-0.067967
36	1	0	2.517577	5.276404	-1.884864
37	1	0	2.274813	3.660869	-2.552208
38	1	0	0.923451	4.796317	-2.472193
39	1	0	1.534153	5.912811	0.346255
40	1	0	0.676327	4.753534	1.349634
41	1	0	-0.094752	5.400076	-0.099344
42	1	0	1.041931	-1.544034	-1.444587
43	1	0	1.932909	-0.050188	-1.295555
44	1	0	3.621469	-1.630175	-1.679719
45	1	0	3.727452	-1.366017	0.038122
46	1	0	0.987600	-3.452719	-0.234522
47	1	0	1.909871	-4.914334	-0.174561

Table S24. The coordinate for the conformer C8 of compound **5** for NMR calculation.

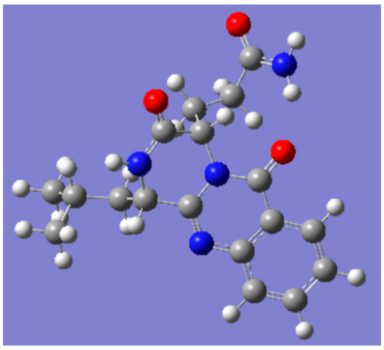

E=-1144.2437043 a.u.

Standard orientation:

Center Number	Atomic Number	Atomic Type	Coordinates (Angstroms) X	Y	Z
1	6	0	3.034693	-4.158045	-0.461293
2	6	0	4.012723	-3.161583	-0.290364
3	6	0	3.634198	-1.866514	0.010366
4	6	0	2.271516	-1.549249	0.140927
5	6	0	1.288874	-2.546411	-0.022096
6	6	0	1.691139	-3.860602	-0.326960
7	6	0	1.858047	-0.180893	0.428862
8	7	0	0.460723	0.017012	0.470240
9	6	0	-0.409654	-1.055678	0.384189
10	7	0	-0.054214	-2.273758	0.145125
11	6	0	-0.034400	1.406536	0.670434
12	6	0	-1.294783	1.447579	1.534758
13	7	0	-2.097768	0.353280	1.469533
14	6	0	-1.890170	-0.818995	0.626064
15	8	0	2.632864	0.751960	0.609700
16	1	0	-2.204067	-1.689673	1.203710
17	6	0	-2.703144	-0.803949	-0.690006
18	6	0	-4.229930	-0.710781	-0.511037
19	6	0	-4.902573	-0.572610	-1.883797
20	6	0	-4.811507	-1.907124	0.255090
21	8	0	-1.551911	2.425178	2.213098
22	1	0	0.733080	1.923406	1.242468
23	6	0	-0.290289	2.159401	-0.658796
24	6	0	0.958730	2.606221	-1.442827
25	6	0	1.604122	3.856762	-0.841580
26	8	0	1.162343	4.970418	-1.079855
27	7	0	2.679332	3.638089	-0.039052
28	1	0	3.338902	-5.172785	-0.696999

29	1	0	5.063015	-3.411038	-0.394877
30	1	0	4.367184	-1.080060	0.145768
31	1	0	0.926377	-4.619750	-0.445507
32	1	0	-2.935269	0.400592	2.033388
33	1	0	-2.367295	0.033038	-1.309806
34	1	0	-2.453601	-1.720766	-1.234966
35	1	0	-4.452404	0.203642	0.054800
36	1	0	-5.985807	-0.462470	-1.782751
37	1	0	-4.530678	0.300143	-2.428191
38	1	0	-4.717192	-1.455443	-2.505014
39	1	0	-5.898924	-1.824714	0.334343
40	1	0	-4.424161	-1.991063	1.274959
41	1	0	-4.589366	-2.849013	-0.257725
42	1	0	-0.905607	1.528299	-1.305244
43	1	0	-0.887539	3.044535	-0.423516
44	1	0	1.677233	1.790702	-1.542897
45	1	0	0.639951	2.890201	-2.447845
46	1	0	2.937675	2.704337	0.251001
47	1	0	3.080068	4.430691	0.438830

Table S25. The coordinate for the conformer C9 of compound **5** for NMR calculation.

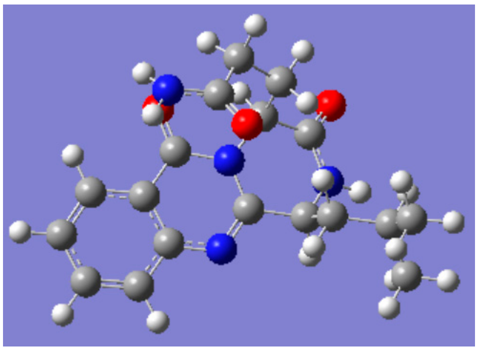

E=-1144.2435726 a.u.

Standard orientation:

Center Number	Atomic Number	Atomic Type	Coordinates (Angstroms) X	Y	Z
1	6	0	-4.099350	-2.722146	0.254596
2	6	0	-4.732378	-1.503174	-0.048861
3	6	0	-3.974844	-0.402304	-0.404120
4	6	0	-2.574697	-0.503964	-0.460587
5	6	0	-1.937500	-1.726379	-0.168827
6	6	0	-2.723016	-2.837597	0.193546
7	6	0	-1.765132	0.659675	-0.809686
8	7	0	-0.370170	0.424162	-0.810795
9	6	0	0.140078	-0.837448	-0.568458
10	7	0	-0.567680	-1.871861	-0.257543
11	6	0	0.501484	1.565871	-1.193479
12	6	0	1.737330	1.113183	-1.969544
13	7	0	2.209680	-0.120317	-1.660209
14	6	0	1.636937	-1.044550	-0.682816
15	8	0	-2.213170	1.768795	-1.070304
16	1	0	1.741552	-2.048400	-1.096355
17	6	0	2.314430	-1.020912	0.708183
18	6	0	3.839266	-1.229016	0.694493
19	6	0	4.388730	-1.095865	2.121407
20	6	0	4.251153	-2.575355	0.082003
21	8	0	2.272503	1.855336	-2.776003
22	1	0	-0.094118	2.170011	-1.875193
23	6	0	0.979248	2.465232	-0.027346
24	6	0	-0.113311	3.087202	0.846811
25	6	0	-0.633838	2.135749	1.922083
26	8	0	0.104739	1.369579	2.522921
27	7	0	-1.960232	2.254969	2.216843
28	1	0	-4.698687	-3.582753	0.534200

29	1	0	-5.813786	-1.429736	-0.006073
30	1	0	-4.437545	0.547158	-0.647520
31	1	0	-2.220675	-3.772287	0.415555
32	1	0	3.047168	-0.398215	-2.153001
33	1	0	2.074678	-0.084157	1.218121
34	1	0	1.841942	-1.812500	1.299887
35	1	0	4.288875	-0.426658	0.094235
36	1	0	5.478956	-1.185025	2.134676
37	1	0	4.126503	-0.130902	2.563695
38	1	0	3.984910	-1.877269	2.773864
39	1	0	5.336476	-2.704866	0.120398
40	1	0	3.956322	-2.676384	-0.967385
41	1	0	3.800773	-3.410946	0.628614
42	1	0	1.670306	1.918700	0.617130
43	1	0	1.556375	3.262435	-0.502568
44	1	0	0.327307	3.929367	1.393096
45	1	0	-0.924306	3.491570	0.239717
46	1	0	-2.593787	2.686468	1.562782
47	1	0	-2.350538	1.606835	2.884748

Table S26. The coordinate for the conformer C10 of compound **5** for NMR calculation.

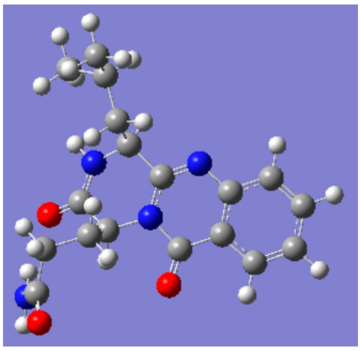

E=-1144.2431876 a.u.

Standard orientation:

Center Number	Atomic Number	Atomic Type	Coordinates (Angstroms) X	Y	Z
1	6	0	5.417479	-0.153039	0.190116
2	6	0	5.149297	-1.530259	0.278378
3	6	0	3.849858	-1.989429	0.161691
4	6	0	2.802205	-1.077601	-0.043927
5	6	0	3.066445	0.304386	-0.135792
6	6	0	4.394281	0.754898	-0.015434
7	6	0	1.425409	-1.554608	-0.155377
8	7	0	0.467401	-0.520165	-0.333507
9	6	0	0.845410	0.799889	-0.441947
10	7	0	2.060364	1.228312	-0.357326
11	6	0	-0.949491	-0.945722	-0.381998
12	6	0	-1.792548	-0.027087	-1.256130
13	7	0	-1.369904	1.250303	-1.380789
14	6	0	-0.230044	1.845643	-0.684933
15	8	0	1.070754	-2.718496	-0.098352
16	1	0	0.234206	2.555856	-1.372216
17	6	0	-0.608876	2.609374	0.613910
18	6	0	-0.997306	4.088128	0.419935
19	6	0	-2.186064	4.300948	-0.527540
20	6	0	-1.277433	4.729142	1.785793
21	8	0	-2.811852	-0.439348	-1.802067
22	1	0	-0.951395	-1.923269	-0.861479
23	6	0	-1.517539	-1.102349	1.053181
24	6	0	-2.988492	-1.547414	1.149840
25	6	0	-3.229085	-2.980014	0.666050
26	8	0	-3.147088	-3.933196	1.424493
27	7	0	-3.538276	-3.099972	-0.653606

28	1	0	6.439025	0.201697	0.282718
29	1	0	5.961766	-2.230751	0.438344
30	1	0	3.613496	-3.045082	0.227397
31	1	0	4.586488	1.819290	-0.089919
32	1	0	-1.965832	1.848933	-1.935615
33	1	0	-1.423977	2.075465	1.114924
34	1	0	0.253667	2.574444	1.285002
35	1	0	-0.125689	4.596247	-0.015050
36	1	0	-2.468624	5.356735	-0.560301
37	1	0	-1.958055	4.012725	-1.558760
38	1	0	-3.066575	3.736576	-0.202948
39	1	0	-1.478426	5.799323	1.685343
40	1	0	-0.429120	4.613848	2.466466
41	1	0	-2.151273	4.274452	2.264873
42	1	0	-0.886776	-1.832116	1.565906
43	1	0	-1.402615	-0.152562	1.584339
44	1	0	-3.271866	-1.532531	2.203708
45	1	0	-3.639729	-0.847559	0.620593
46	1	0	-3.497822	-2.304424	-1.279614
47	1	0	-3.643515	-4.027641	-1.034602

Table S27. The coordinate for the conformer C11 of compound **5** for NMR calculation.

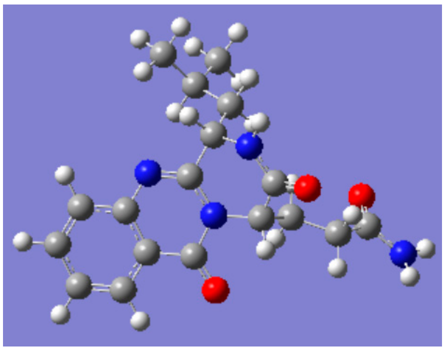

E=-1144.2429465 a.u.

Standard orientation:

Center Number	Atomic Number	Atomic Type	Coordinates (Angstroms) X	Y	Z
1	6	0	-4.965639	-1.549227	-0.721214
2	6	0	-4.325516	-2.797265	-0.819455
3	6	0	-2.984105	-2.911566	-0.502866
4	6	0	-2.265066	-1.779902	-0.085595
5	6	0	-2.902514	-0.526906	0.016200
6	6	0	-4.268738	-0.428010	-0.308911
7	6	0	-0.848086	-1.891018	0.251656
8	7	0	-0.245451	-0.668566	0.642389
9	6	0	-0.971793	0.501894	0.718710
10	7	0	-2.229187	0.605335	0.437997
11	6	0	1.190753	-0.749024	1.000870
12	6	0	1.604713	0.307878	2.019616
13	7	0	0.825969	1.417006	2.090164
14	6	0	-0.255101	1.760175	1.167228
15	8	0	-0.191748	-2.919630	0.217455
16	1	0	-0.996911	2.320458	1.736788
17	6	0	0.252286	2.647678	0.000330
18	6	0	-0.812030	3.254432	-0.931988
19	6	0	-1.779527	4.188205	-0.193202
20	6	0	-0.116057	3.993964	-2.083276
21	8	0	2.591928	0.141614	2.719888
22	1	0	1.315394	-1.713833	1.491689
23	6	0	2.084336	-0.723212	-0.261465
24	6	0	3.510033	-1.208174	0.002530
25	6	0	4.413230	-0.989062	-1.206807
26	8	0	4.217416	-0.112633	-2.032412
27	7	0	5.481136	-1.836353	-1.290185

28	1	0	-6.018606	-1.465138	-0.970708
29	1	0	-4.884804	-3.668444	-1.143147
30	1	0	-2.465290	-3.860823	-0.568633
31	1	0	-4.749935	0.539907	-0.225014
32	1	0	1.148265	2.127510	2.734019
33	1	0	0.828536	3.462567	0.458101
34	1	0	0.969470	2.072650	-0.592898
35	1	0	-1.399469	2.435490	-1.361146
36	1	0	-2.481955	4.648946	-0.893755
37	1	0	-2.375036	3.658486	0.553657
38	1	0	-1.241623	5.001021	0.308895
39	1	0	-0.848037	4.400287	-2.787020
40	1	0	0.552162	3.333947	-2.643820
41	1	0	0.483040	4.832791	-1.711759
42	1	0	1.616242	-1.368326	-1.007735
43	1	0	2.120210	0.280195	-0.688261
44	1	0	3.948433	-0.664478	0.845786
45	1	0	3.503030	-2.264643	0.290816
46	1	0	5.677993	-2.527785	-0.586106
47	1	0	6.163773	-1.682728	-2.015923

Table S28. The coordinate for the conformer C12 of compound **5** for NMR calculation.

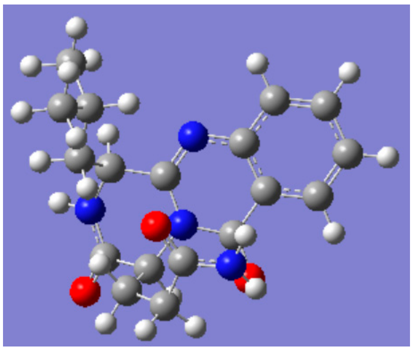

E=-1144.2429299 a.u.

Standard orientation:

Center Number	Atomic Number	Atomic Type	Coordinates (Angstroms) X	Y	Z
1	6	0	4.003019	2.430755	-0.327753
2	6	0	4.587186	1.152651	-0.267477
3	6	0	3.792671	0.025340	-0.368957
4	6	0	2.403575	0.159586	-0.530040
5	6	0	1.815770	1.437786	-0.600755
6	6	0	2.638233	2.575892	-0.495012
7	6	0	1.553827	-1.024300	-0.620798
8	7	0	0.173871	-0.746739	-0.760524
9	6	0	-0.286304	0.553349	-0.870697
10	7	0	0.459400	1.605869	-0.798910
11	6	0	-0.727972	-1.917662	-0.919197
12	6	0	-1.892862	-1.625168	-1.864502
13	7	0	-2.301792	-0.332848	-1.914804
14	6	0	-1.765916	0.769862	-1.115271
15	8	0	1.958610	-2.178483	-0.571522
16	1	0	-1.817289	1.662573	-1.738625
17	6	0	-2.578171	1.021932	0.182468
18	6	0	-2.338680	2.377204	0.873967
19	6	0	-2.808870	3.564846	0.023700
20	6	0	-3.033141	2.378476	2.242904
21	8	0	-2.426345	-2.525831	-2.490491
22	1	0	-0.121718	-2.685007	-1.397197
23	6	0	-1.312689	-2.502935	0.390288
24	6	0	-0.304207	-2.905239	1.468946
25	6	0	0.193126	-1.722847	2.297424
26	8	0	-0.533438	-0.786186	2.596935
27	7	0	1.478247	-1.820094	2.742279

28	1	0	4.631187	3.312189	-0.247289
29	1	0	5.660417	1.053565	-0.144849
30	1	0	4.218058	-0.970878	-0.332325
31	1	0	2.173700	3.553782	-0.552195
32	1	0	-3.110599	-0.155482	-2.495258
33	1	0	-3.639406	0.938861	-0.085990
34	1	0	-2.370379	0.222015	0.896411
35	1	0	-1.262629	2.486690	1.044348
36	1	0	-2.658111	4.507318	0.558004
37	1	0	-2.264619	3.646130	-0.920986
38	1	0	-3.878027	3.488989	-0.207143
39	1	0	-2.860393	3.321796	2.769601
40	1	0	-2.661817	1.568988	2.876575
41	1	0	-4.117567	2.255487	2.139463
42	1	0	-2.032930	-1.810791	0.829108
43	1	0	-1.876681	-3.385445	0.078337
44	1	0	-0.813798	-3.566017	2.180274
45	1	0	0.524185	-3.474672	1.045094
46	1	0	2.129450	-2.449504	2.300983
47	1	0	1.858695	-1.037059	3.252580

Table S29. The coordinate for the conformer C13 of compound **5** for NMR calculation.

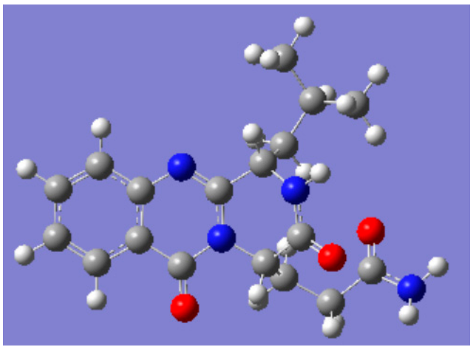

E=-1144.2425119 a.u.

Standard orientation:

Center Number	Atomic Number	Atomic Type	Coordinates (Angstroms) X	Y	Z
1	6	0	-5.487627	0.679675	-0.366516
2	6	0	-5.532100	-0.725868	-0.373604
3	6	0	-4.370753	-1.453646	-0.189221
4	6	0	-3.150052	-0.786701	0.004209
5	6	0	-3.100544	0.622494	0.015310
6	6	0	-4.292420	1.348035	-0.174066
7	6	0	-1.915998	-1.547153	0.185568
8	7	0	-0.760259	-0.744148	0.352264
9	6	0	-0.833046	0.632718	0.375307
10	7	0	-1.920743	1.312575	0.221791
11	6	0	0.523694	-1.468526	0.488339
12	6	0	1.539824	-0.705842	1.331050
13	7	0	1.376497	0.634312	1.411620
14	6	0	0.447085	1.418204	0.597645
15	8	0	-1.829384	-2.765043	0.192191
16	1	0	0.136614	2.273312	1.199015
17	6	0	1.051879	1.932540	-0.737639
18	6	0	1.887950	3.229368	-0.654744
19	6	0	1.040563	4.457670	-0.296028
20	6	0	3.121259	3.119851	0.254007
21	8	0	2.437597	-1.310140	1.902948
22	1	0	0.296985	-2.376071	1.047115
23	6	0	1.055994	-1.886336	-0.904522
24	6	0	2.401713	-2.616489	-0.885609
25	6	0	3.603166	-1.674556	-0.825892
26	8	0	3.572419	-0.550924	-1.306501
27	7	0	4.741067	-2.209476	-0.294491

28	1	0	-6.401996	1.246117	-0.512142
29	1	0	-6.477050	-1.236862	-0.523806
30	1	0	-4.374943	-2.537461	-0.190923
31	1	0	-4.242910	2.431017	-0.161930
32	1	0	2.101035	1.124544	1.916939
33	1	0	1.668625	1.139956	-1.168699
34	1	0	0.221010	2.114901	-1.426335
35	1	0	2.261326	3.381458	-1.675687
36	1	0	1.636599	5.372702	-0.362236
37	1	0	0.186010	4.567644	-0.969707
38	1	0	0.647796	4.407260	0.725077
39	1	0	3.777170	3.984417	0.116118
40	1	0	3.703850	2.220599	0.038119
41	1	0	2.848689	3.110819	1.316582
42	1	0	0.297198	-2.540667	-1.337062
43	1	0	1.139152	-1.009828	-1.549637
44	1	0	2.443170	-3.353926	-0.079921
45	1	0	2.506310	-3.176446	-1.822257
46	1	0	4.669883	-2.968295	0.365044
47	1	0	5.510957	-1.571820	-0.152605

Table S30. The coordinate for the conformer C14 of compound **5** for NMR calculation.

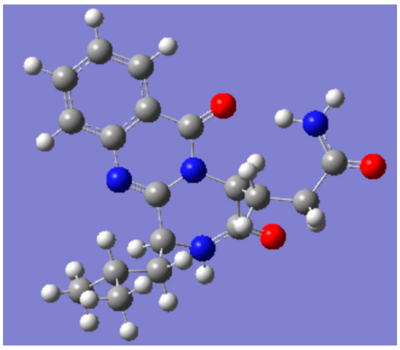

E=-1144.242472 a.u.

Standard orientation:

Center Number	Atomic Number	Atomic Type	Coordinates (Angstroms) X	Y	Z
1	6	0	-3.907904	3.257637	0.085235
2	6	0	-2.818571	4.116496	0.317909
3	6	0	-1.526877	3.628770	0.248745
4	6	0	-1.307815	2.274312	-0.053586
5	6	0	-2.396309	1.409704	-0.287270
6	6	0	-3.705025	1.922959	-0.213845
7	6	0	0.047171	1.745381	-0.133865
8	7	0	0.129732	0.373376	-0.447970
9	6	0	-1.008525	-0.384789	-0.653951
10	7	0	-2.214451	0.073922	-0.592057
11	6	0	1.503758	-0.176972	-0.584449
12	6	0	1.566971	-1.396294	-1.501513
13	7	0	0.408407	-2.081564	-1.683214
14	6	0	-0.840453	-1.858441	-0.960466
15	8	0	1.066850	2.402786	0.048147
16	1	0	-1.655400	-2.102997	-1.641661
17	6	0	-0.948041	-2.767131	0.292752
18	6	0	-2.318766	-2.835461	0.991224
19	6	0	-3.411475	-3.414827	0.083268
20	6	0	-2.192269	-3.660019	2.280101
21	8	0	2.618093	-1.715043	-2.029961
22	1	0	2.084088	0.594927	-1.088048
23	6	0	2.137652	-0.466422	0.803236
24	6	0	3.665459	-0.619725	0.804632
25	6	0	4.557203	0.623657	0.729901
26	8	0	5.769229	0.496542	0.816367
27	7	0	3.963532	1.838964	0.588421

28	1	0	-4.919140	3.647720	0.140053
29	1	0	-2.993863	5.161171	0.550624
30	1	0	-0.670174	4.269233	0.422074
31	1	0	-4.534371	1.249584	-0.398242
32	1	0	0.487060	-2.908909	-2.259943
33	1	0	-0.662929	-3.777169	-0.028794
34	1	0	-0.185670	-2.457960	1.015197
35	1	0	-2.616431	-1.818068	1.267543
36	1	0	-4.357889	-3.499862	0.624525
37	1	0	-3.599695	-2.787847	-0.791154
38	1	0	-3.145496	-4.419028	-0.266740
39	1	0	-3.143721	-3.696219	2.817937
40	1	0	-1.445178	-3.238586	2.959457
41	1	0	-1.898120	-4.693099	2.064472
42	1	0	1.835734	0.331044	1.486150
43	1	0	1.699088	-1.385915	1.202377
44	1	0	3.965318	-1.124415	1.727630
45	1	0	3.987384	-1.276572	-0.006761
46	1	0	2.972047	1.995289	0.457771
47	1	0	4.576342	2.637495	0.524492

Table S31. The coordinate for the conformer C15 of compound **5** for NMR calculation.

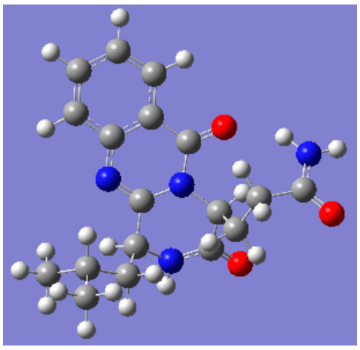

E=-1144.2423185 a.u.

Standard orientation:

Center Number	Atomic Number	Atomic Type	Coordinates (Angstroms) X	Y	Z
1	6	0	-3.004467	3.915670	0.240173
2	6	0	-1.749108	4.549880	0.256559
3	6	0	-0.599277	3.812539	0.043120
4	6	0	-0.689009	2.428852	-0.184332
5	6	0	-1.944880	1.791032	-0.207711
6	6	0	-3.106450	2.556363	0.008215
7	6	0	0.517131	1.635175	-0.388761
8	7	0	0.295396	0.248719	-0.536704
9	6	0	-0.991346	-0.258393	-0.623125
10	7	0	-2.066867	0.440698	-0.470702
11	6	0	1.491227	-0.625689	-0.691161
12	6	0	1.239173	-1.780173	-1.663445
13	7	0	-0.051007	-2.188336	-1.784282
14	6	0	-1.173386	-1.724572	-0.973250
15	8	0	1.650912	2.100303	-0.422622
16	1	0	-2.055488	-1.740477	-1.614063
17	6	0	-1.438937	-2.658005	0.236881
18	6	0	-2.736749	-2.422188	1.031090
19	6	0	-3.994563	-2.619816	0.174937
20	6	0	-2.761456	-3.352144	2.252293
21	8	0	2.162577	-2.290570	-2.270406
22	1	0	2.256065	-0.010119	-1.160345
23	6	0	2.025715	-1.178441	0.653739
24	6	0	2.771945	-0.177788	1.557174
25	6	0	4.197406	0.092765	1.070362
26	8	0	5.107035	-0.682932	1.320978
27	7	0	4.369034	1.234796	0.352989

28	1	0	-3.903024	4.500838	0.407290
29	1	0	-1.685014	5.617513	0.436344
30	1	0	0.379543	4.277231	0.050401
31	1	0	-4.067276	2.054985	-0.017695
32	1	0	-0.197152	-2.981279	-2.394903
33	1	0	-1.448840	-3.681320	-0.160666
34	1	0	-0.581923	-2.613395	0.915167
35	1	0	-2.736776	-1.387957	1.392233
36	1	0	-4.896823	-2.504843	0.782322
37	1	0	-4.061474	-1.891746	-0.636533
38	1	0	-4.023308	-3.624682	-0.262454
39	1	0	-3.656405	-3.181374	2.856982
40	1	0	-1.891889	-3.196958	2.898037
41	1	0	-2.765289	-4.405263	1.950404
42	1	0	1.190095	-1.603084	1.215650
43	1	0	2.701163	-2.006070	0.421333
44	1	0	2.205053	0.747028	1.677912
45	1	0	2.876449	-0.632453	2.544516
46	1	0	3.578327	1.783580	0.043026
47	1	0	5.276089	1.403894	-0.054208

Table S32. The coordinate for the conformer C16 of compound **5** for NMR calculation.

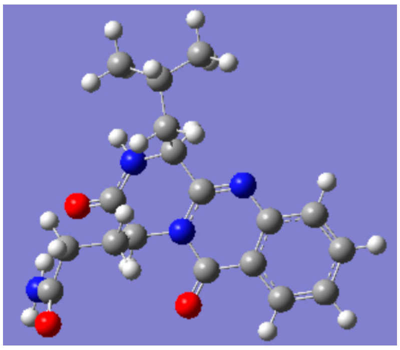

E=-1144.2418739 a.u.

Standard orientation:

Center Number	Atomic Number	Atomic Type	Coordinates (Angstroms) X	Y	Z
1	6	0	5.246711	-1.142858	0.155390
2	6	0	4.734640	-2.452028	0.135352
3	6	0	3.372945	-2.659008	0.008511
4	6	0	2.506007	-1.559826	-0.099277
5	6	0	3.015031	-0.245194	-0.082931
6	6	0	4.403100	-0.052019	0.046592
7	6	0	1.065003	-1.770431	-0.220356
8	7	0	0.308310	-0.569274	-0.292687
9	6	0	0.918788	0.665702	-0.304408
10	7	0	2.191665	0.859452	-0.209135
11	6	0	-1.162379	-0.729223	-0.346803
12	6	0	-1.831694	0.394923	-1.125823
13	7	0	-1.187637	1.582290	-1.155786
14	6	0	0.050840	1.904828	-0.447908
15	8	0	0.506365	-2.852338	-0.252468
16	1	0	0.629556	2.563665	-1.096527
17	6	0	-0.170255	2.619878	0.913369
18	6	0	-0.387621	4.149090	0.859635
19	6	0	0.878275	4.903521	0.430264
20	6	0	-1.598511	4.582614	0.021527
21	8	0	-2.911307	0.217392	-1.682760
22	1	0	-1.341259	-1.646345	-0.906046
23	6	0	-1.742983	-0.903509	1.081159
24	6	0	-3.268589	-1.091493	1.167673
25	6	0	-3.755870	-2.415258	0.573366
26	8	0	-3.826264	-3.431083	1.246884
27	7	0	-4.100198	-2.365868	-0.742220

28	1	0	6.316100	-0.986493	0.255070
29	1	0	5.408076	-3.298041	0.219928
30	1	0	2.950339	-3.656880	-0.007681
31	1	0	4.783698	0.963130	0.054922
32	1	0	-1.665755	2.319253	-1.655324
33	1	0	-1.021778	2.149731	1.415363
34	1	0	0.709650	2.428386	1.533935
35	1	0	-0.597695	4.438306	1.897629
36	1	0	0.729628	5.984218	0.508961
37	1	0	1.735477	4.638880	1.055162
38	1	0	1.153489	4.694663	-0.608599
39	1	0	-1.808686	5.645060	0.172172
40	1	0	-2.503509	4.028059	0.285730
41	1	0	-1.421257	4.455492	-1.052810
42	1	0	-1.248678	-1.772508	1.521490
43	1	0	-1.461472	-0.035896	1.685653
44	1	0	-3.541395	-1.115422	2.224227
45	1	0	-3.789443	-0.247836	0.708557
46	1	0	-3.928282	-1.538808	-1.301487
47	1	0	-4.362746	-3.226840	-1.196783

© 2020 by the authors. Licensee MDPI, Basel, Switzerland. This article is an open access article distributed under the terms and conditions of the Creative Commons Attribution (CC BY) license (http://creativecommons.org/licenses/by/4.0/).
